# Dynamic cellular programs of human cardiac allograft rejection revealed by spatial transcriptomics

**DOI:** 10.1038/s44161-026-00849-9

**Published:** 2026-08-10

**Authors:** Kaushik Amancherla, Angela M. Taravella Oill, Xavier Bledsoe, Arianna L. Williams, Nelson Chow, Anna J. Smith, Melissa A. Farrow, Shilin Zhao, Quanhu Sheng, David W. Bearl, Alexander N. Perez, Robert D. Hoffman, Jonathan N. Menachem, Hasan K. Siddiqi, Douglas M. Brinkley, Evan D. Mee, Niran Hadad, Vineet Agrawal, Jeffrey Schmeckpepper, Aniket Rali, Stacy Tsai, Eric Farber-Eger, Quinn S. Wells, Jane E. Freedman, Nathan R. Tucker, Jeffrey M. Spraggins, Kelly H. Schlendorf, Eric R. Gamazon, Nicholas Banovich, Ravi V. Shah

**Affiliations:** 1 Vanderbilt Translational and Clinical Cardiovascular Research Center, Vanderbilt University School of Medicine, Nashville, TN USA; 2 Vanderbilt University School of Medicine, Nashville, TN USA; 3 Division of Bioinnovation and Genome Sciences, The Translational Genomics Research Institute, Phoenix, AZ USA; 4 Department of Medicine, Vanderbilt University Medical Center, Nashville, TN USA; 5 Department of Biostatistics, Vanderbilt University Medical Center, Nashville, TN USA; 6 Department of Pediatrics, Vanderbilt University Medical Center, Nashville, TN USA; 7 Department of Pathology, Microbiology, and Immunology, Vanderbilt University Medical Center, Nashville, TN USA; 8 The State University of New York Upstate Medical University, Syracuse, NY USA; 9 Department of Anesthesiology, Vanderbilt University Medical Center, Nashville, TN USA

**Keywords:** Transcriptomics, Heart failure

## Abstract

Allograft rejection following solid-organ transplantation is a major cause of graft dysfunction and mortality. Current diagnostic approaches rely on histology, which exhibits wide diagnostic variability and lacks clinically relevant molecular phenotyping. Here we leverage image-based spatial transcriptomics at subcellular resolution in longitudinal human cardiac biopsies to characterize transcriptional heterogeneity in 62 adult and pediatric heart transplant recipients during and following histologically diagnosed rejection. Across 28 cell types, we identified significant differences in abundance in immune and parenchymal cells across different classes of rejection. We observed broad overlap in transcriptional states across rejection severity and significant heterogeneity within rejection grades. Responders and nonresponders to augmented therapies had distinct transcriptomic profiles, with nonresponders exhibiting baseline T cell hyperactivation and tissue remodeling genes. We also identified cell-specific genes linked to long-term outcomes after heart transplant. These results underscore the importance of subtyping cellular states during rejection to stratify immune–cardiac interactions relevant to short- and long-term outcomes.

## Abstract (web summary)

Using spatial transcriptomics on longitudinal human cardiac biopsies, Amancherla, Oill and colleagues reveal marked cellular and molecular heterogeneity in transplanted hearts undergoing rejection, identify gene expression profiles linked to therapy response and outcomes, and show that acute rejection signatures are associated with chronic rejection development.

## Main

Solid-organ transplantation is the definitive treatment for end-stage organ failure. However, allograft rejection remains common early after transplantation (up to ~40% of recipients experience rejection within 1 year post-transplant) and results in long-term graft failure and death^1–6^. Diagnosis of acute rejection and its response to therapy relies upon tissue histology^7,8^, subject to interobserver variability^9^ that impacts precision of immunomodulatory therapies and potential for over- or under-immunosuppression^10–12^ with downstream clinical consequence. Heart transplantation (HT) represents a paradigmatic setting for this process: acute rejection affects ~20% of recipients within the first-year post-HT^6^ and places patients at risk for chronic rejection phenotypes (cardiac allograft vasculopathy (CAV)) that limit long-term survival^13–15^. Rejection severity is graded by invasive endomyocardial biopsy (EMB)^10,16,17^, although some evidence suggests that histology alone does not precisely phenotype rejection: (1) clinical presentation varies dramatically within the same grade of histologic rejection^18^; and (2) response to antirejection therapy is heterogenous, including lack of response in some patients^19,20^. Moreover, there remains a substantial gap connecting acute rejection and its resolution to long-term outcomes (for example, CAV). Given the routine performance of EMB for histologic rejection surveillance post-HT, HT represents a unique setting to study the impact of deeper spatially resolved molecular profiling that may inform precision approaches.

Here, we use spatial transcriptomics at subcellular resolution to delineate the molecular underpinnings of acute rejection in hearts from 62 children and adults following HT. We studied transcriptional profiles across rejection class and type as well as serial biopsies within the same patients during and after rejection therapy to map histologic–transcriptional concordance and dynamic responses to treatment. Finally, we explored the relation of cell-specific gene expression signatures to CAV development to assess the ability of spatially resolved genomics to highlight cellular programs relevant to long-term outcomes. Our overall goal was to create a longitudinal biopsy resource in HT to study tissue-based, spatially resolved genomics as an actionable molecular phenotype in acute rejection after solid organ transplantation.

## Results

### Study participants

**Fig. 1:**
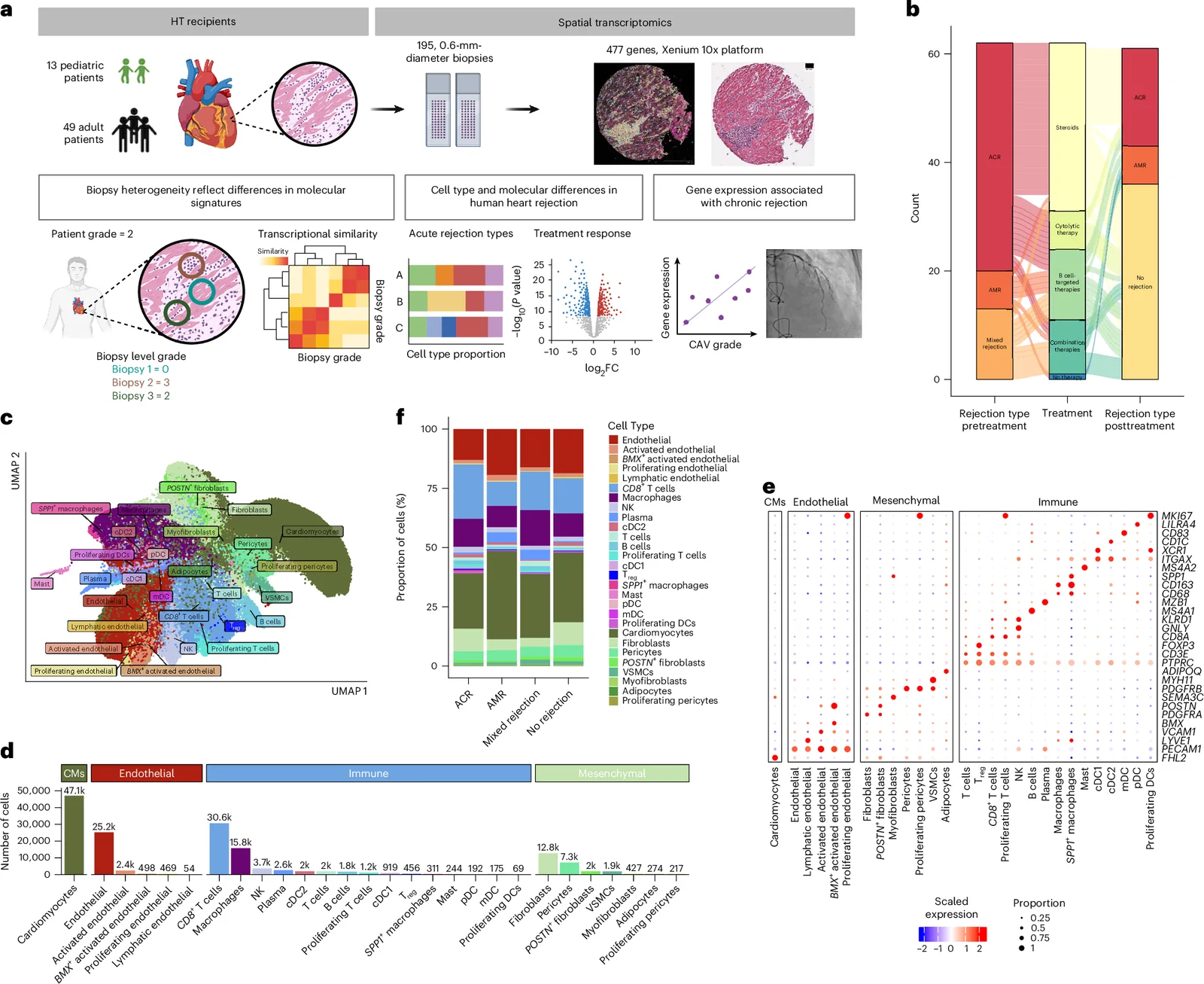
High-resolution spatial mapping of the cellular landscape in human cardiac allograft rejection. **a**, Study overview. **b**, Alluvial plot demonstrating rejection grading at the time of diagnosis in the left column, immunomodulatory therapies in the middle column, and biopsy grading following therapies in the right column. B cell-targeted therapies include combinations of intravenous immunoglobulin, plasmapheresis, rituximab, bortezomib and eculizumab. Cytolytic therapies represent thymoglobulin. Combination therapies correspond to combinations of cytolytic and B cell-targeted therapies. **c**, UMAP representing cell types identified. A total of 10,343,560 high quality transcripts were measured among 162,638 cells. **d**, Bar plot demonstrating the 28 cell types identified and their respective cell numbers measured. **e**, Dot plot heatmap of select marker genes used for annotated cell types in the dataset. See Extended Data Fig. 1 for the expanded version. **f**, Cell type composition across rejection types. DCs, dendritic cells; CMs, cardiomyocytes. Schematics in **a** created in BioRender; Banovich, N. https://biorender.com/n62l518 (2025). Source data

Our study overview is outlined in Fig. 1a. We obtained longitudinal EMBs from 49 adult and 13 pediatric HT recipients. Median recipient age at time of HT was 52 years (27% female) for adults and 6 years (62% female) for children. Complete donor/recipient characteristics are presented in Table 1. Biopsies were collected at a median of 313 and 407 days post-HT for EMBs during rejection and following immunomodulatory therapies, respectively (Supplementary Fig. 1). Among biopsies demonstrating rejection, 67.7% comprised of acute cellular rejection (ACR), 11.3% antibody-mediated rejection (AMR) and 21% mixed rejection (both ACR and AMR). Consistent with clinical practice, we observed a broad range of immunomodulatory therapies (corticosteroids, 50%; cytolytic T cell-targeted therapies, 11.3%; B cell-targeted therapies, 21%; combination cytolytic/B cell therapy, 16.1%; no therapy, 1.6%; Fig. 1b).

**Table 1:** Baseline characteristics of the cohort

Characteristic	Overall, *N* = 62^a^	Age group	
		Adult, *N* = 49^a^	Pediatric, *N* = 13^a^
Age at transplant (years)	47 (25–58)	52 (44–62)	6 (2–12)
Recipient gender			
Female	21 (34%)	13 (27%)	8 (62%)
Male	41 (66%)	36 (73%)	5 (38%)
Class 1 DSAs	22 (35%)	14 (29%)	8 (62%)
Class 2 DSAs	40 (65%)	27 (55%)	13 (100%)
Deceased	20 (32%)	16 (33%)	4 (31%)
CAV grade			
0	38 (61%)	31 (63%)	7 (54%)
1	17 (27%)	14 (29%)	3 (23%)
2	3 (4.8%)	1 (2.0%)	2 (15%)
3	4 (6.5%)	3 (6.1%)	1 (7.7%)
Donor death type			
DBD	58 (94%)	45 (92%)	13 (100%)
DCD	4 (6.5%)	4 (8.2%)	0 (0%)
Donor HCV NAT status			
Negative	50 (81%)	37 (76%)	13 (100%)
Positive	12 (19%)	12 (24%)	0 (0%)
Donor age (years)^b^	27 (20, 36)	30 (23, 37)	7 (2, 10)
Donor gender^b^			
Female	17 (28%)	13 (27%)	4 (33%)
Male	43 (72%)	35 (73%)	8 (67%)

^a^Median (IQR); *n* (%). ^b^Missing data on two individuals due to transfer of care from another transplant center.

DSA, donor-specific antibody; DBD, donation after brain death; DCD, donation after circulatory death; HCV, hepatitis C virus; NAT, nucleic acid test.

### Compositional and spatial landscape of acute rejection highlights functionally relevant cell types post-HT

**Extended Data Fig. 1:**
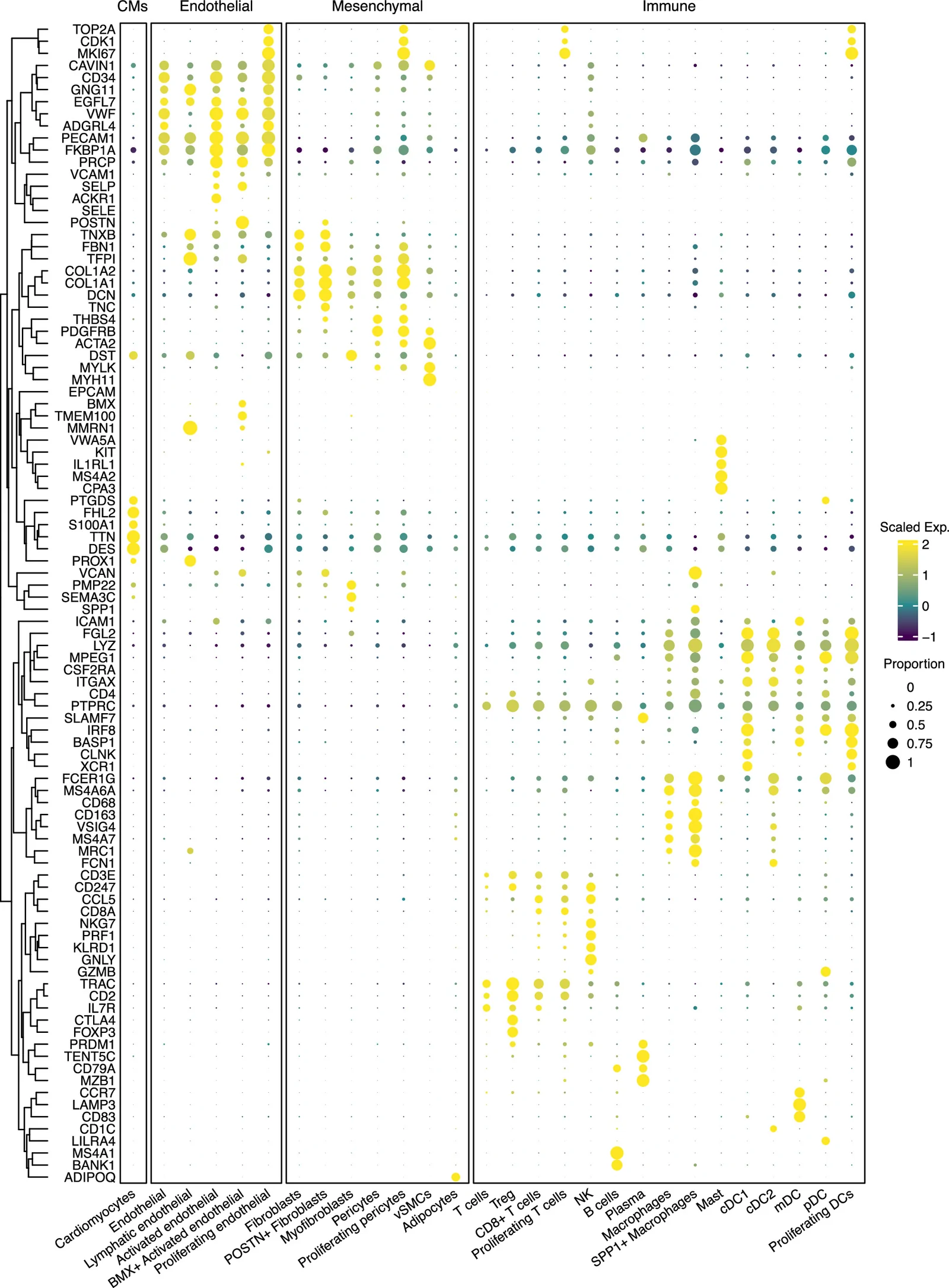
Marker genes for all cell types. Dotplot heatmap of a combination the top five expressed marker genes for each cell type and canonical marker genes to demonstrate cell type annotation in our data set. Source data

**Extended Data Fig. 2:**
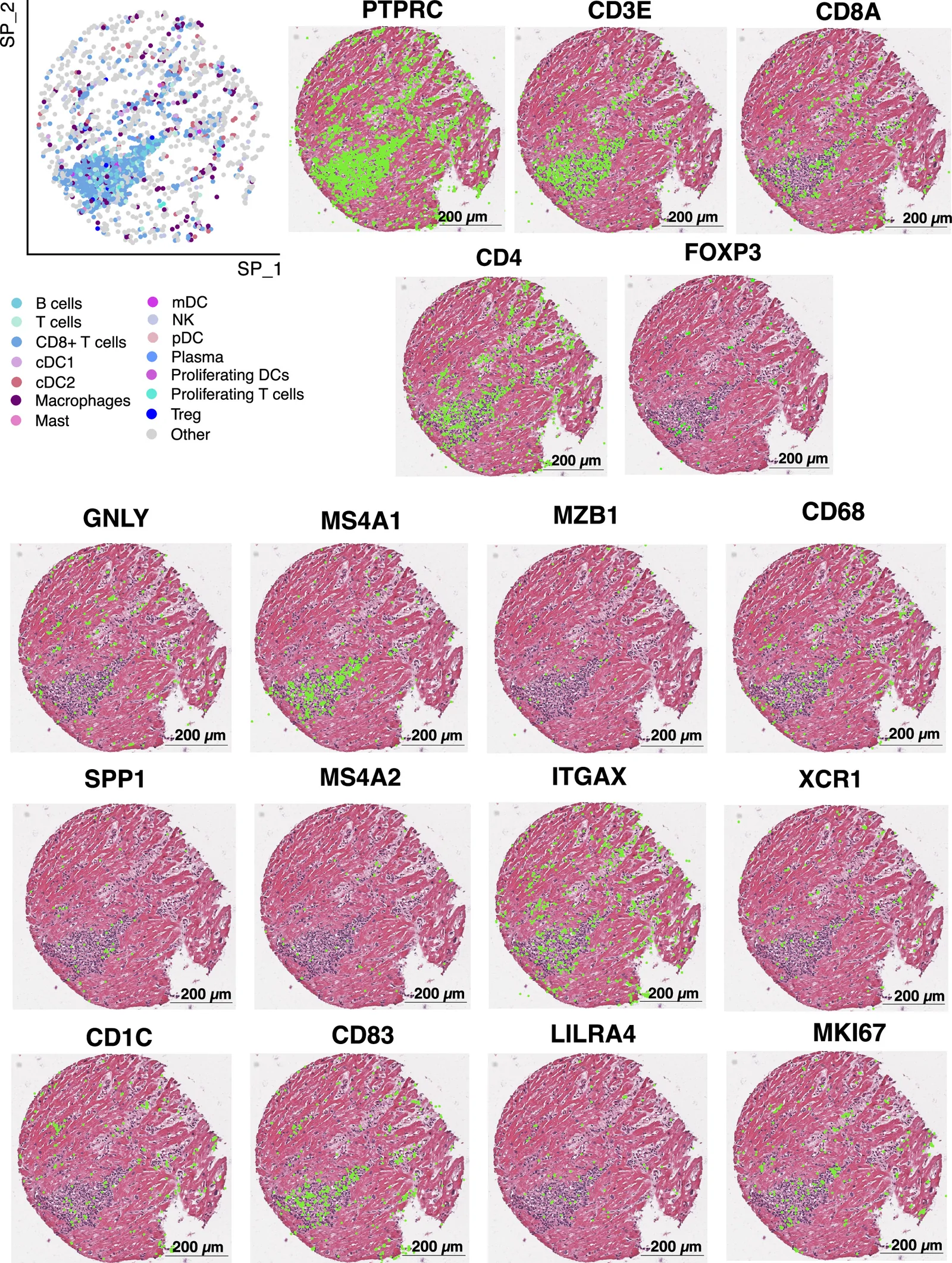
Spatial organization of immune cells in an ACR patient biopsy. Spatial dim plot for a biopsy from an ACR patient. Non-immune cell types are colored in grey, immune cell types are colored based on the key provided. Overlaid transcripts (green) on H&Es for set of genes used for cell type annotation of immune cells. Transcripts are colored in green.

**Extended Data Fig. 3:**
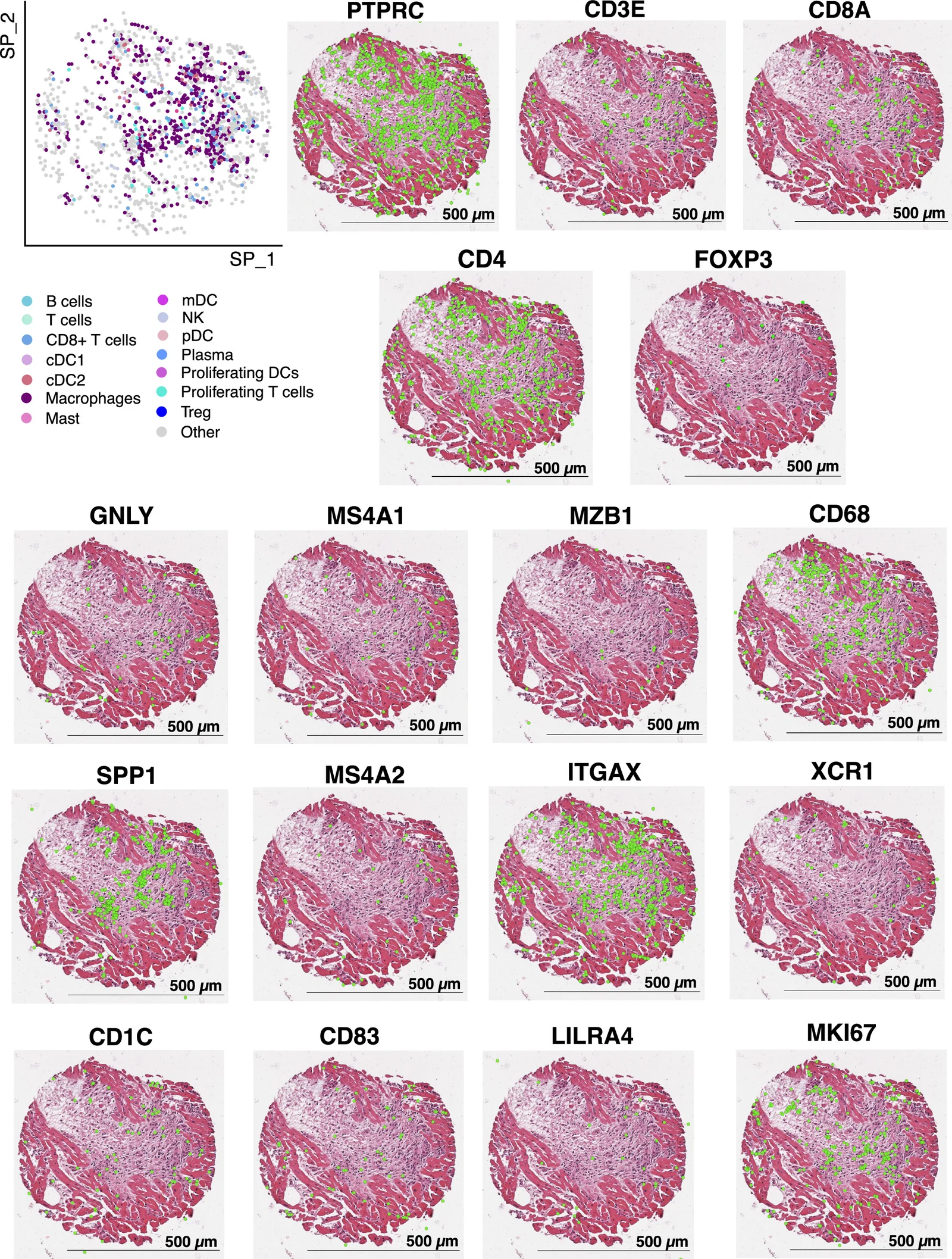
Spatial organization of immune cells in an AMR patient biopsy. Spatial dim plot for a biopsy from an AMR patient. Non-immune cell types are colored in grey, immune cell types are colored based on the key provided. Overlaid transcripts (green) on H&Es for set of genes used for cell type annotation of immune cells. Transcripts are colored in green.

**Extended Data Fig. 4:**
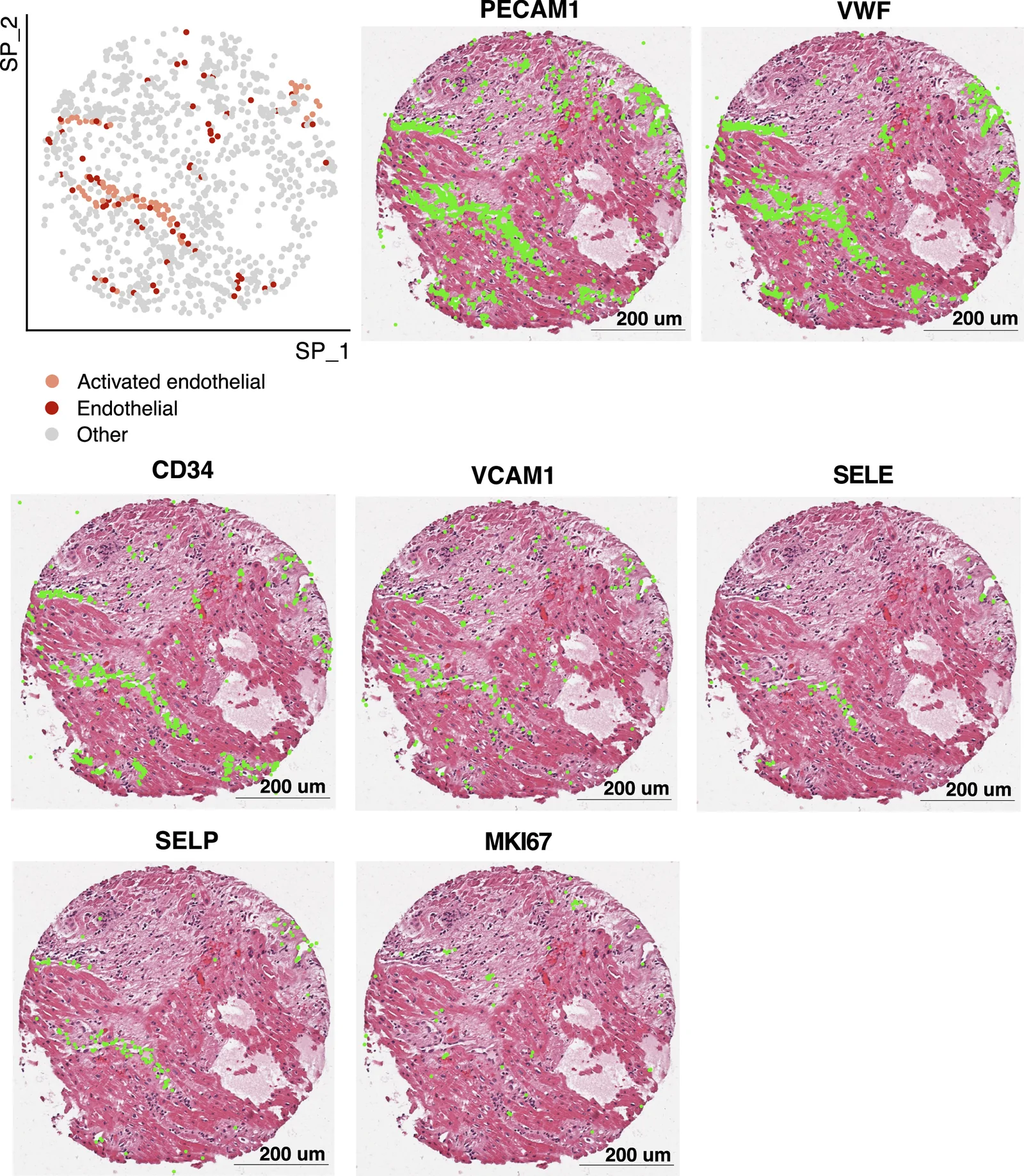
Spatial organization of endothelial cells in an ACR patient biopsy. Spatial dim plot for a biopsy from an ACR patient. Non-endothelial cell types are colored in grey, endothelial cell types are colored based on the key provided. Overlaid transcripts (green) on H&Es for set of genes used for cell type annotation of endothelial cells. Transcripts are colored in green.

**Extended Data Fig. 5:**
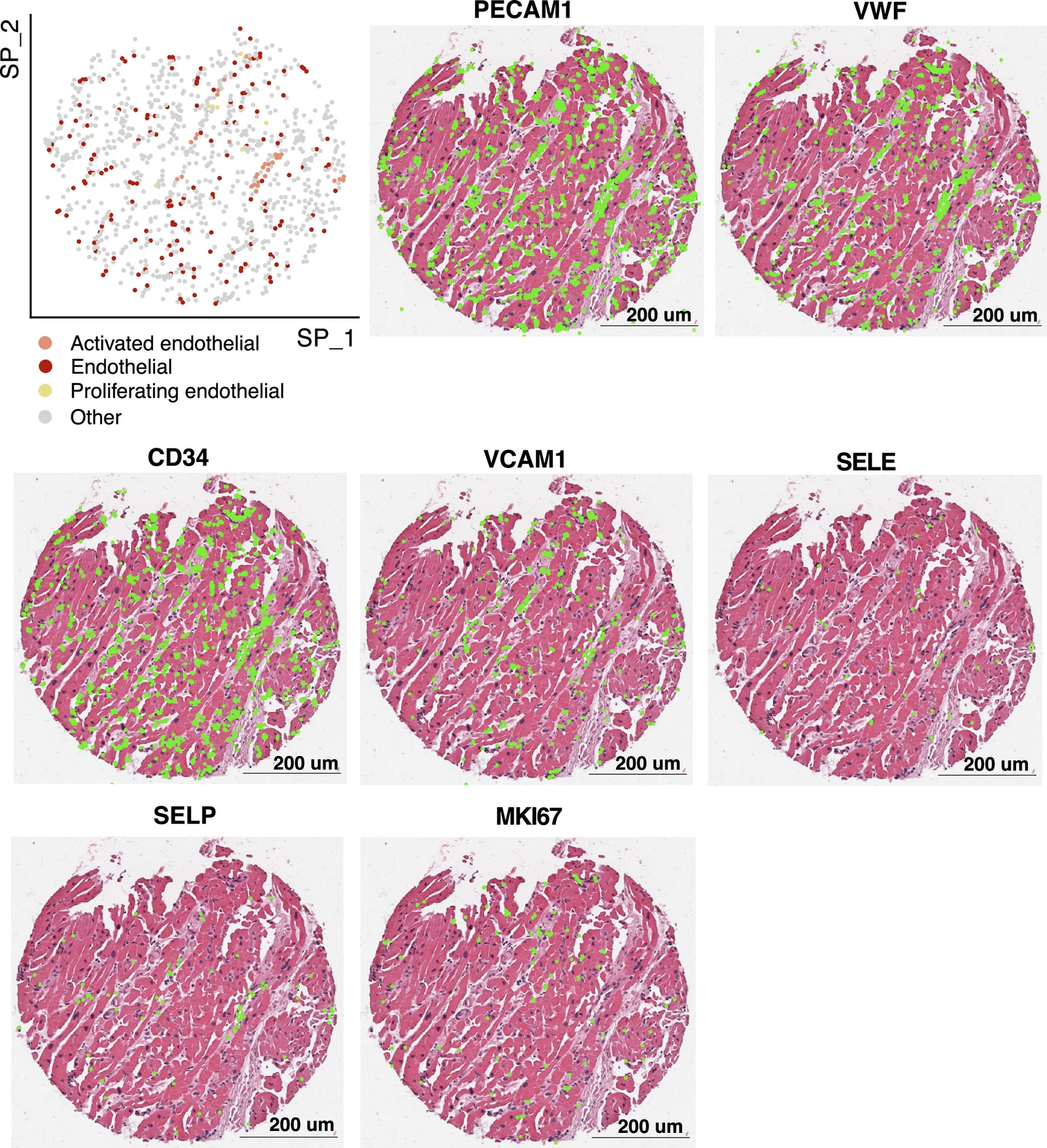
Spatial organization of endothelial cells in an AMR patient biopsy. Spatial dim plot for a biopsy from an AMR patient. Non-endothelial cell types are colored in grey, endothelial cell types are colored based on the key provided. Overlaid transcripts (green) on H&Es for set of genes used for cell type annotation of endothelial cells. Transcripts are colored in green.

**Extended Data Fig. 6:**
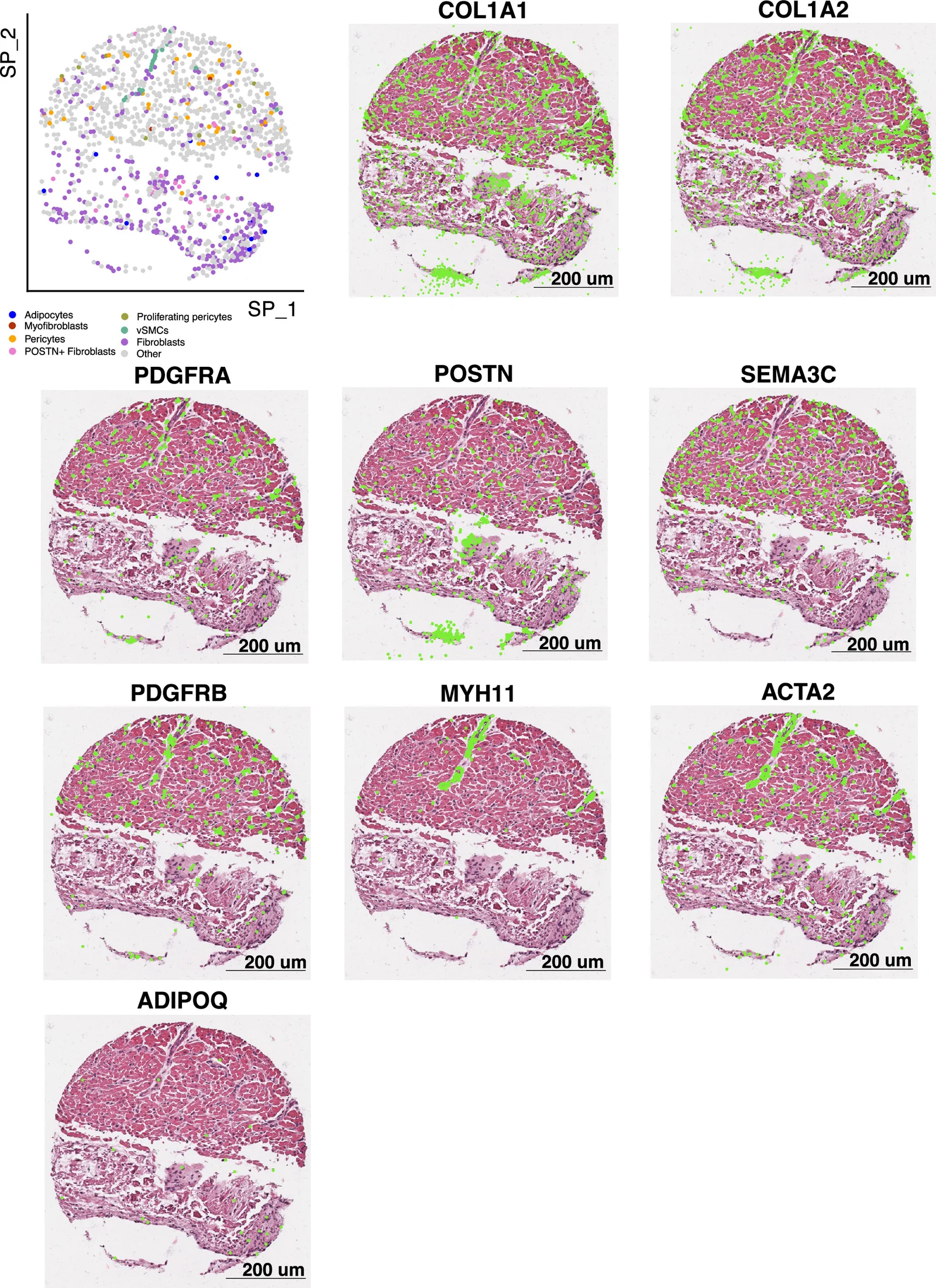
Spatial organization of mesenchymal cells in an ACR patient biopsy. Spatial dim plot for biopsy from an ACR patient. Non-mesenchymal cell types are colored in grey, mesenchymal cell types are colored based on the key provided. Overlaid transcripts (green) on H&Es for set of genes used for cell type annotation of mesenchymal cells. Transcripts are colored in green.

**Extended Data Fig. 7:**
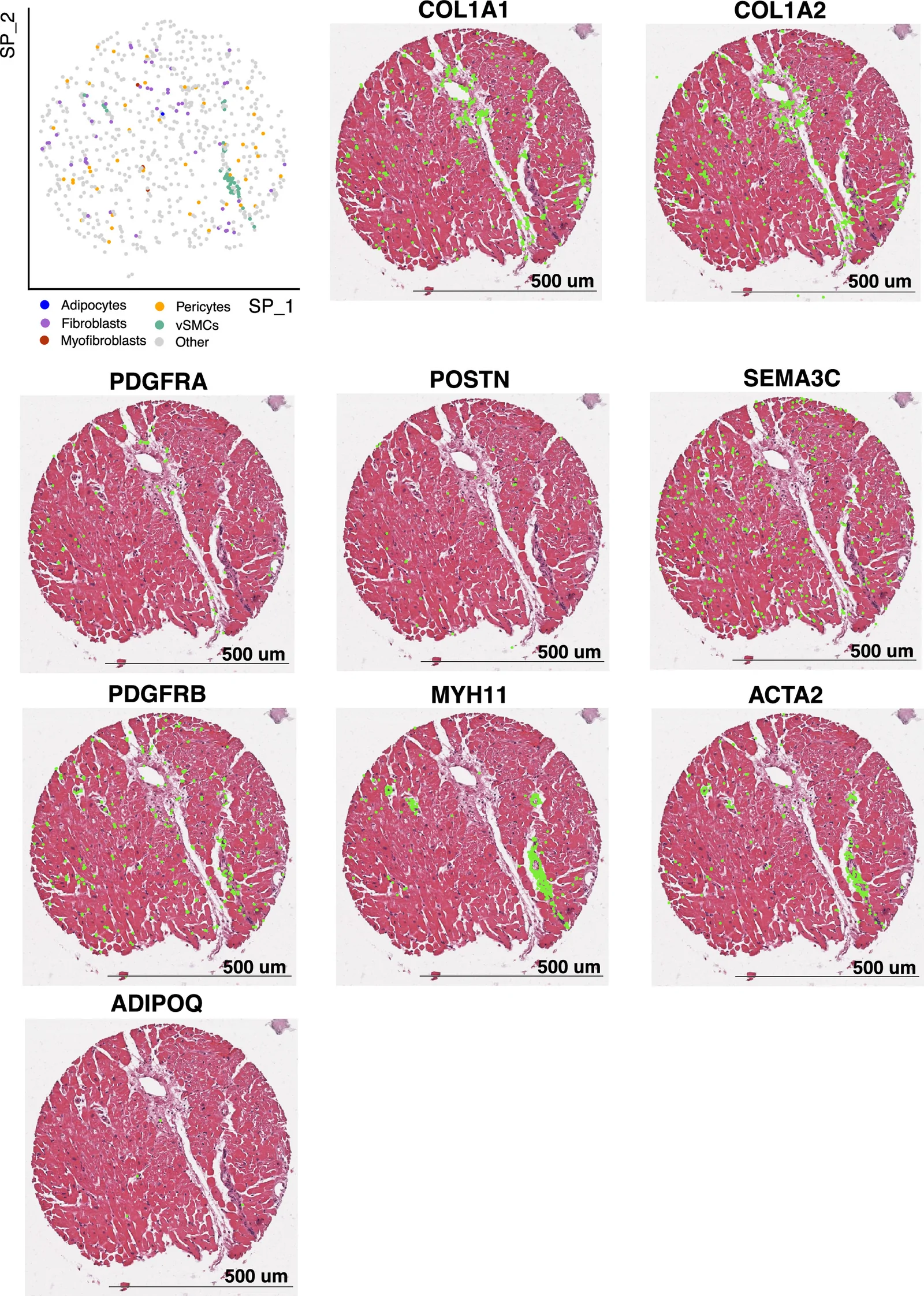
Spatial organization of mesenchymal cells in an AMR patient biopsy. Spatial dim plot for biopsy from an AMR patient. Non-mesenchymal cell types are colored in grey, mesenchymal cell types are colored based on the key provided. Overlaid transcripts (green) on H&Es for set of genes used for cell type annotation of mesenchymal cells. Transcripts are colored in green.

**Extended Data Fig. 8:**
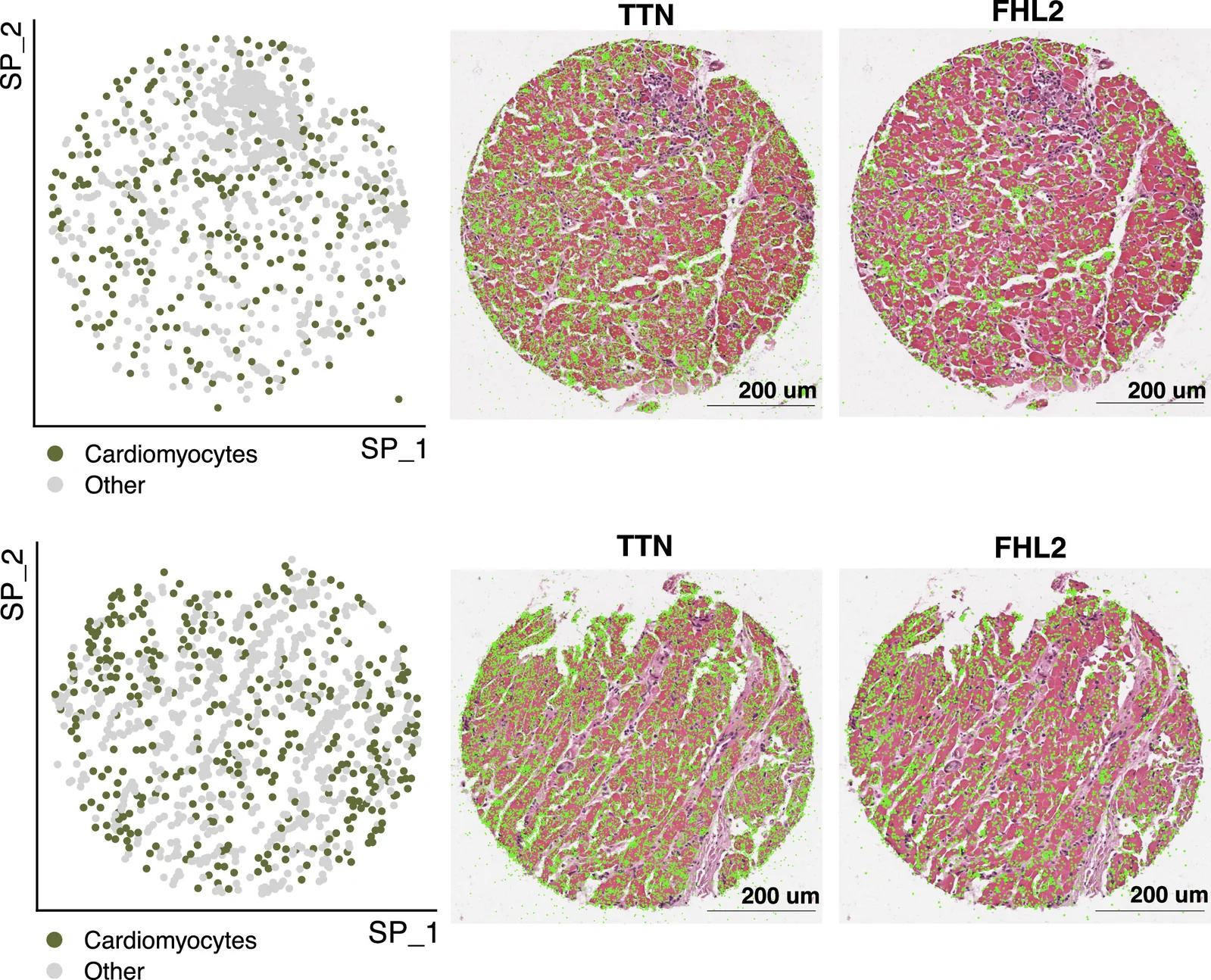
Spatial organization of cardiomyocytes in an ACR and AMR patient biopsy. Spatial dim plots for an ACR (top) and AMR (bottom) patient biopsy. Overlaid transcripts (green) on H&Es for set of genes used for cell type annotation of cardiomyocytes.

**Extended Data Fig. 9:**
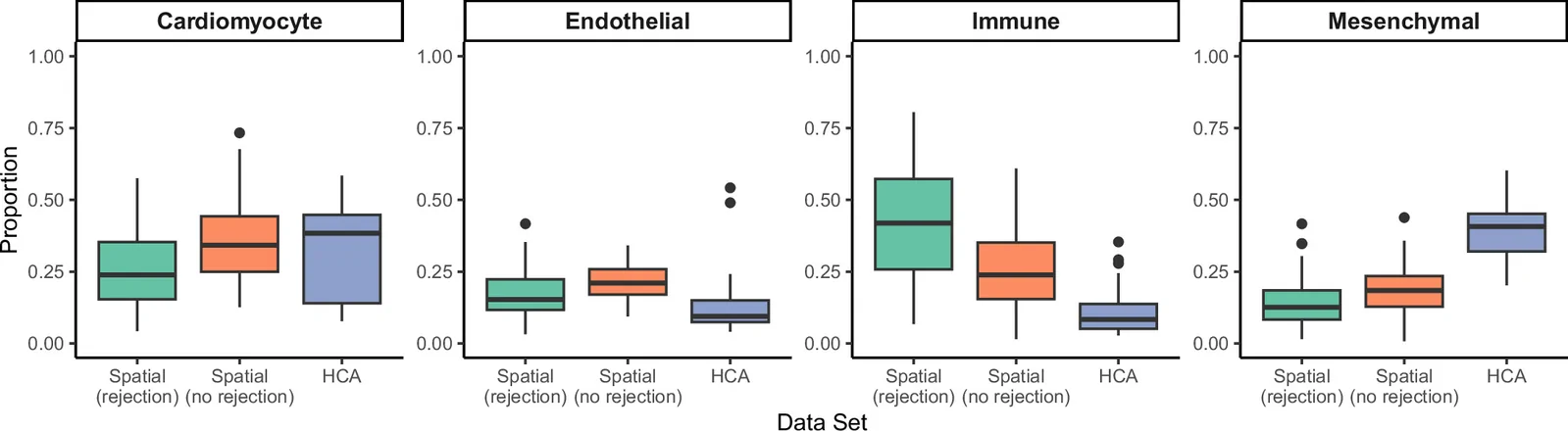
Comparison of cellular proportions in rejecting and non-rejecting allografts relative to the human Heart Cell Atlas reference. Boxplots comparing proportion of cell types by compartment recovered from our study (divided by the presence or absence of acute rejection) in comparison to the Heart Cell Atlas (HCA; 25 adult donors, 14 females and 11 males). Source data

To better understand cell types driving allograft rejection, we evaluated compositional differences between the three types of rejection and following antirejection therapies. Across all samples, we measured 10,343,560 high-quality transcripts among 162,638 cells comprising 28 cell types (Fig. 1c–e and Extended Data Figs. 1–8). Beyond canonical cardiac cell types, we identified *POSTN*^+^ activated fibroblasts, myofibroblasts, *BMX*^+^ activated endothelial cells, *SPP1*^+^ macrophages and proliferating pericytes, among others. Compared with prior single-cell/single-nucleus RNA sequencing (RNA-seq) studies of the heart, we recover more immune and endothelial cells (Extended Data Fig. 9). Across cell types, B cells, cardiomyocytes, *CD4*^+^ and *CD8*^+^ T cells, dendritic cells (cDC1 and cDC2), endothelial cells, fibroblasts, lymphatic endothelial cells, plasma cells, proliferating pericytes, proliferating T cells and *SPP1*^+^ macrophages were significantly differentially abundant in acute rejection (false discovery rate (FDR) <0.1; Fig. 1f and Supplementary Table 1).

Overall, we observed cell types in rejection with plausible pathophysiologic connection related to its acute and chronic complications post-HT. In ACR, *CD8*^+^ T cells, proliferating T cells, B cells, cDC1, fibroblasts and *SPP1*^+^ macrophages accounted for the highest cell type proportion. Of note, fibroblasts and *SPP1*^+^ macrophages may mediate cardiac fibrosis^21,22^, a precursor finding of later CAV and allograft failure^23,24^. Moreover, *SPP1*^+^ macrophages have been implicated as key drivers of atrial fibrillation^25^, associated with acute rejection^26,27^. By contrast, we observed the highest proportions of endothelial cells, plasma cells, cardiomyocytes, macrophages and proliferating pericytes in individuals with AMR, with a trend toward increased activated and proliferating endothelial cells. Given the link between AMR, microvascular injury, and long-term CAV and graft failure, the high abundance of antibody-producing plasma cells, endothelial cells and proliferating pericytes—all relevant to endothelial and coronary vascular injury—support the clinical epidemiology. We then performed immunohistochemistry against CD3, CD8 and CD68 for orthogonal validation (Supplementary Fig. 2 and Supplementary Table 2). Comparing five nonrejecting EMBs with five ACR and five AMR EMBs, we observed significantly higher expression in ACR of all three markers in comparison with nonrejecting biopsies. Consistent with compositional changes observed in our spatial transcriptomics data, CD3 and CD8 expression was significantly higher in ACR compared with AMR.

**Fig. 2:**
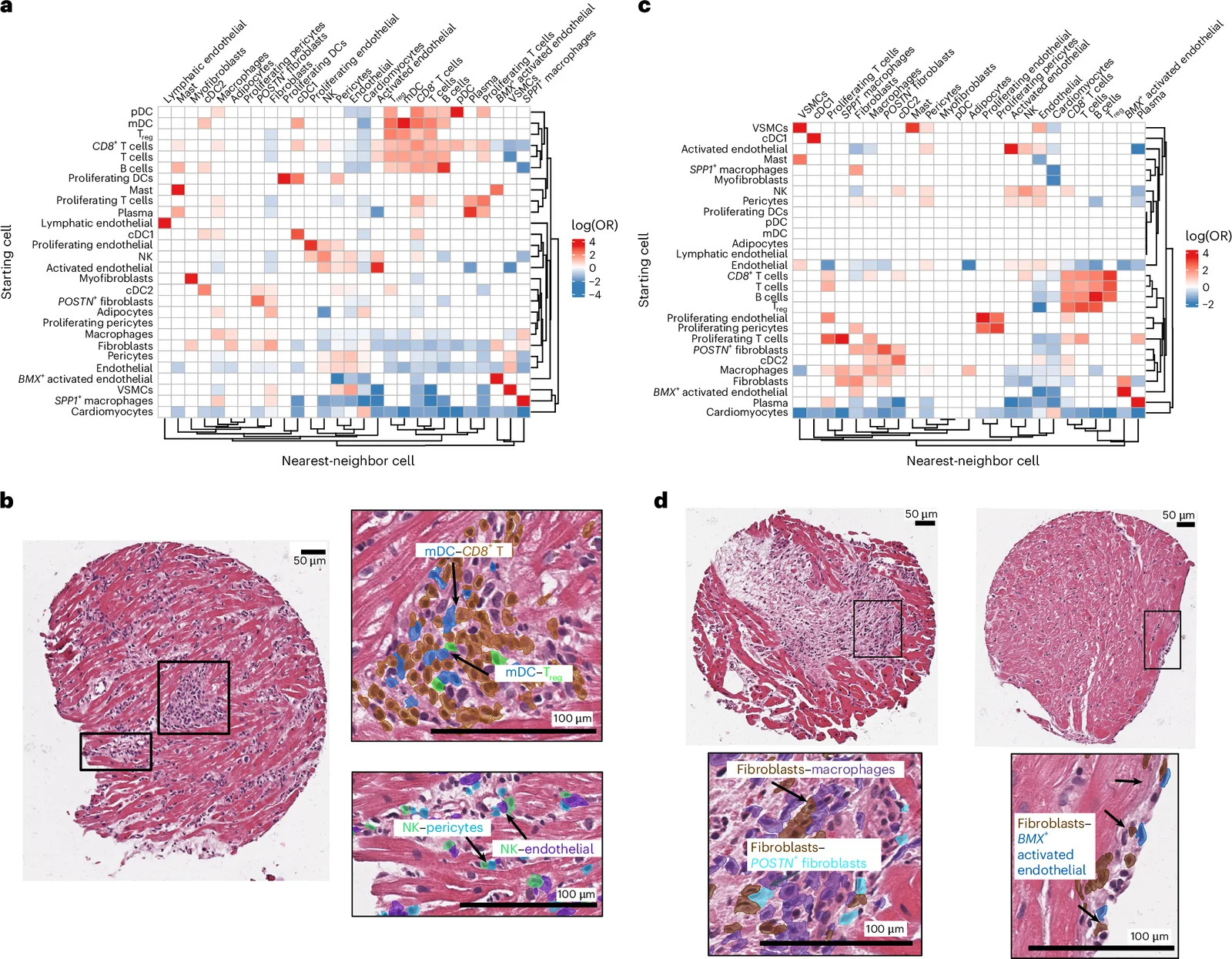
Spatial colocalization in acute cardiac allograft rejection. **a**–**d**, Cell proximity results across ACR pretreatment samples (**a** and **b**) and AMR pretreatment samples (**c** and **d**). The heatmaps (**a** and **c**) show the log odds ratio (OR) where positive values (red) represent cell-type pairs that are proximal to each other while a negative value (blue) represents cell-type pairs that are less likely to be found near each other. Nonsignificant results (FDR >0.05) are coded as 0. Highlighted examples of proximal cell types are overlaid on H&E stainings for ACR (**b**) and AMR (**d**) biopsies. Cells are colored by cell types, and arrows show specific significant source cell–neighbor pairs. In the ACR biopsy, the following source cell–neighbor pairs are highlighted: mDC–*CD8*^+^ T cell, mDC–T_reg_, NK–endothelial and NK–pericyte. In the AMR biopsies, the following source cell–neighbor pairs are highlighted: macrophage–fibroblast, proliferating pericyte–fibroblast, *POSTN*^+^ fibroblast–fibroblast, and *BMX*^+^ activated endothelial–fibroblast. Source data

We next sought to establish spatial localization of cell types in rejection. We calculated the pairwise likelihood of cell types being in close proximity. In both AMR and ACR, we find all T cell subsets tend to be in close proximity to one another. In ACR, we find that this immune localization also includes plasmacytoid dendritic cells (pDCs) and migratory dendritic cells (mDCs) (Fig. 2a,b). Furthermore, in ACR, natural killer (NK) cells closely localize with endothelial, activated endothelial cells, and pericytes (Fig. 2b). In AMR, we find an additional localization of macrophage and fibroblast subtypes, where fibroblasts are also in close proximity to *BMX*^+^ activated endothelial cells and proliferating pericytes (Fig. 2c,d). These results are consistent with observed focal fibrosis near damaged vasculature^10–13,28^.

### Substantial transcriptional heterogeneity exists within and across histologic rejection grade

**Fig. 3:**
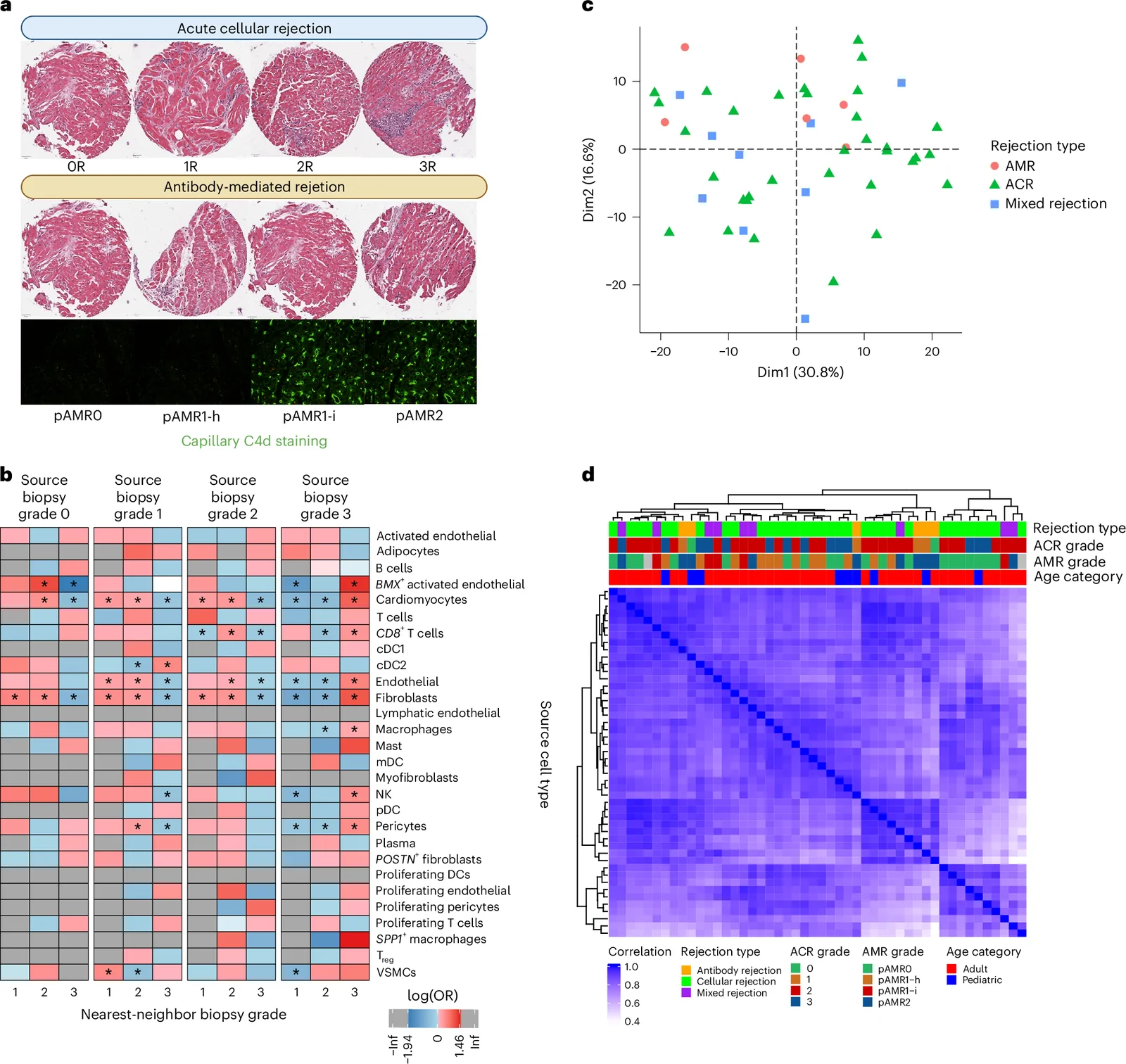
Substantial transcriptional heterogeneity exists within and across histologic rejection grades. **a**, Representative examples of rejection types and severity. Clinically meaningful rejection grades warranting augmented immunomodulatory therapies include 2R and 3R within ACR and pAMR1-i and pAMR2 within AMR. Diagnosis of AMR required the presence of C4d deposition (bottom portion of **a**) in ≥50% of capillaries within the biopsy sample. **b**, Heatmap showing transcriptional similarity within and between biopsy grades. A positive log(OR) (red) represents biopsy grade pairs that are more transcriptionally similar, while negative log(OR) (blue) represents biopsy grade pairs that are less transcriptionally similar; extreme values of −Inf and Inf correspond to odds ratios of 0 and infinity, respectively, where biopsy grade pairs exhibit zero or absolute overlap in transcriptionally similar cell states. Asterisks denote significant (FDR <0.05) biopsy grade pair associations. We excluded the nearest-neighbor biopsy grade 0 for visualization purposes, as none of the comparisons was significant. **c**, PCA plot of all three rejection types based on pseudobulk gene expression demonstrates lack of clear clustering by histological rejection diagnosis. **d**, Cluster dendrogram assessing similarity across rejection types. These results support that rejection is molecularly heterogeneous across biological class (for example, ACR versus AMR) and within the same severity of rejection grading (for example, grade 2R cellular rejection), despite similar histological appearance. Schematics in **a** created in BioRender; Hu, R. https://biorender.com/y78m858 (2026). Source data

Histologic rejection grades drive clinical therapies for ACR post-HT^7,10–12^. However, despite similar appearances on histology, clinical presentation is heterogenous, ranging from asymptomatic incidental findings on routine surveillance biopsy to cardiogenic shock. Similarly, therapeutic response is varied without predictability of histological response to immunomodulatory therapies. Therefore, we next sought to understand concordance between these clinically utilized histologic grades and transcriptional phenotypes within rejection. Clinical grading of EMBs to determine acute rejection grade was carried out according to International Society for Heart and Lung Transplantation (ISHLT) society standards^7,10^ by an experienced cardiovascular transplant pathologist (R.D.H.) using whole-slide biopsies—clinical ‘gold standard’— distinct from those used for spatial profiling (tissue microarrays (TMAs) constructed from biopsies). Notably, clinical grading requires multiple biopsies from the same patient as pathologic abnormalities in EMBs are often patchy, such that different fragments or local regions from the same biopsy set may show different degrees or patterns of injury. While this heterogeneity adds complexity to clinical rejection diagnosis, it provides an opportunity to understand the degree to which local remodeling drives local cellular states. We generated high-quality hematoxylin and eosin (H&E) images from samples after spatial transcriptomic analysis, enabling matched histology and molecular analysis. Using matched criteria to clinical grading, we generated a biopsy-level grade for each pretreatment ACR TMA sample (Fig. 3a). For ACR cases, this local TMA-level assessment was particularly important because inflammatory injury may be patchy. TMA-level assessments for AMR remained concordant with the EMB-level diagnosis, consistent with the diffuse C4d positivity used in our institutional assessment of AMR. Next, we examined the transcriptional similarity of cells within and between biopsy grades. In general, we found cells from ACR grade ≤2R biopsies were more similar to other lower-grade biopsies, with grade 3R biopsies having a distinct molecular profile (Fig. 3b). This association was particularly pronounced for fibroblasts, cardiomyocytes and endothelial cells which showed significant transcriptional similarity across nearly all low-grade biopsies and a distinct profile in grade 3R biopsies. Overall, these data suggest that the molecular state of cells are heavily influenced by the local remodeling regardless of the overall patient grade.

Turning our attention to clinical (that is, patient level) grading, we quantified transcriptional heterogeneity across individuals. We created a pseudobulk expression profile for each individual and calculated the principal components (PCs) of gene expression to reduce dimensionality (Supplementary Figs. 3 and 4). The PC projections using histologically determined grading according to clinical standards did not clearly separate the type or grade of rejection (Fig. 3c and Supplementary Fig. 5), suggesting underlying heterogeneity unexplained by histology alone. This was reinforced by hierarchical clustering, which demonstrated broad overlap in gene expression profile across rejection types, grades and patient ages (Fig. 3d). Together, these results suggest that molecular heterogeneity (masked by histology alone) may potentially explain the substantial variability in clinical presentation and therapeutic response that exists even within a given rejection grade.

### Spatial transcriptomics resolves functionally relevant patterns of gene expression between ACR and AMR

**Fig. 4:**
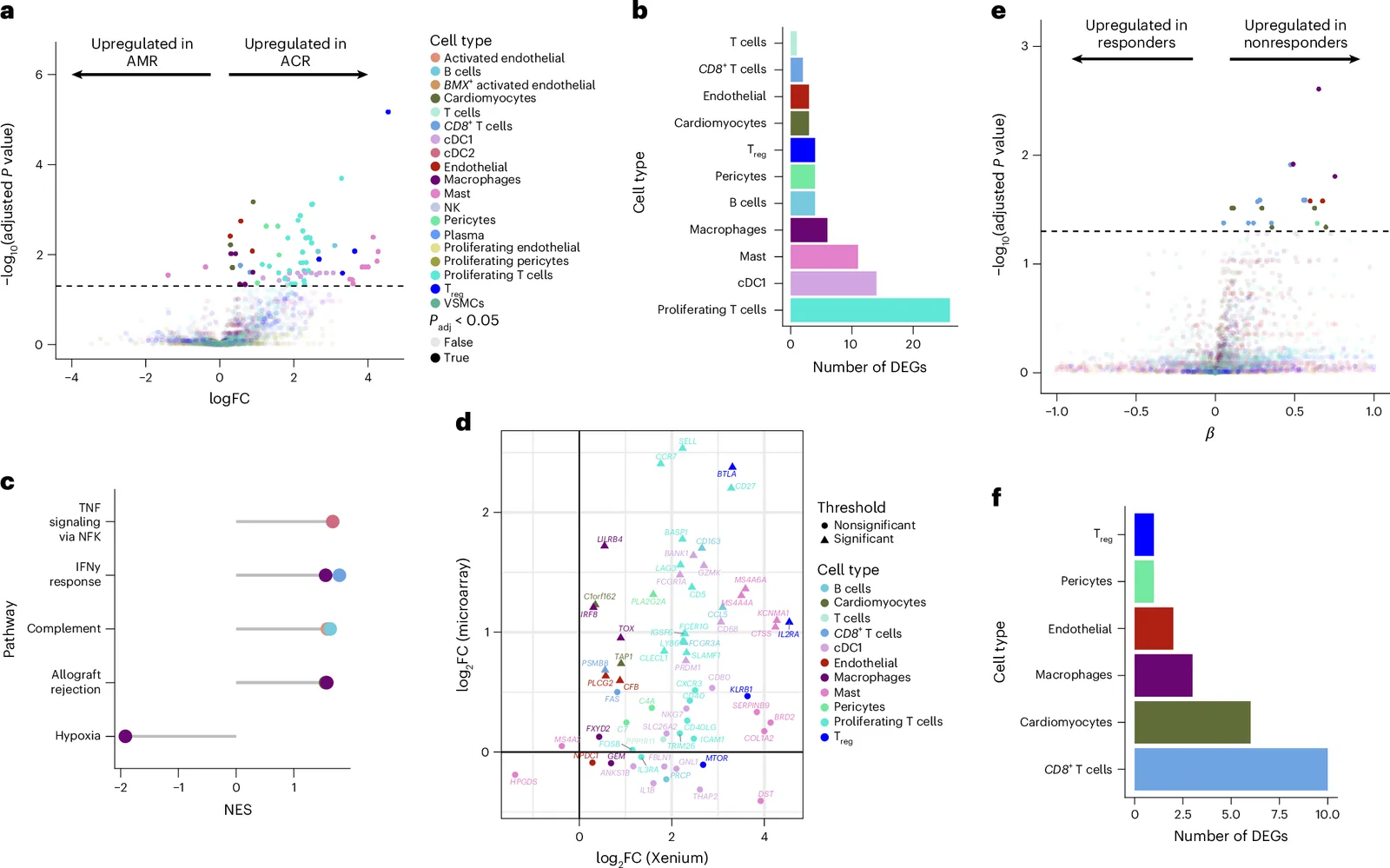
Cell-type-resolved transcriptomics defines molecular signatures of acute rejection type and therapyresistance. **a**, Volcano plot representing genes with significant differences in expression between ACR and AMR. A positive log_2_FC means upregulated in ACR. A negative log_2_FC means upregulated in AMR. The color of each dot represents its corresponding cell type. Transparency reflects adjusted *P* values, with transparent dots below the dashed black line representing those with adjusted *P* values ≥0.05. Differential expression was assessed using a two-sided Welch’s *t*-test, and *P* values were adjusted for multiple comparisons using the Benjamini–Hochberg FDR. Supplementary Table 3 contains the full DEG results. **b**, Bar plot demonstrating the number of DEGs among cell types. **c**, GSEA depicting results of hallmark pathways that showed significance at an FDR <0.1. A positive normalized enrichment score (NES) means enriched in ACR, while a negative NES means enriched in AMR. Supplementary Table 4 contains full results of the GSEA. **d**, Overlap between DEGs (adjusted *P* value <0.05) in Xenium compared with DEGs (Benjamini–Hochberg-corrected *P* value <0.05 and |log_2_FC| >1.5) in a publicly available EMB microarray dataset (GSE150059) comparing 76 patients with ACR and 179 patients with AMR. Scatter plot of the 65 unique genes that were differentially expressed between ACR and AMR in our study (Xenium) and tested in the EMB dataset. The *x* axis represents log_2_FC in the Xenium dataset, while the *y* axis represents log_2_FC in the microarray dataset. Significant genes from the EMB microarray data are depicted by triangles. Genes depicted as circles were not significant in the EMB microarray data. Colors represent cell type specificity of DEGs from the Xenium dataset. When more than one cell type–gene pair was significant in the Xenium dataset, the cell type with the lowest adjusted *P* value is depicted. **e**, Volcano plot representing genes with significant difference in expression between responders and nonresponders to immunomodulatory therapies during ACR. A positive log_2_FC means upregulated in persistent ACR (nonresponders). A negative log_2_FC means upregulated in those whose ACR resolved (responders). The color of each dot represents its corresponding cell type. Transparency reflects adjusted *P* values, with transparent dots below the dashed black line representing those with adjusted *P* values ≥0.05. Differential expression was assessed using a linear model with TMA cellular grade included as a covariate, with significance based on a two-sided *t*-test; *P* values were adjusted for multiple comparisons using the Benjamini–Hochberg FDR. Supplementary Table 7 contains the full DEG results. **f**, Bar plot demonstrating the number of DEGs per cell type between ACR responders and nonresponders.

Clinical treatments for ACR and AMR largely differ, with ongoing uncertainty around selection of optimal therapies across rejection^7,10^. We hypothesized that cell-specific differences in gene expression between ACR and AMR (Supplementary Table 3) may shed light on clinical–hemodynamic differences between them^10^. Distinguishing ACR and AMR, we identified 78 significant gene–cell type pairs representing 70 unique genes and spanning 11 cell types (FDR <0.05; Fig. 4a). The cell type with the largest number of differentially expressed genes (DEGs) was proliferating T cells (26 DEGs), followed by cDC1 cells (14 DEGs) and mast cells (11 DEGs; Fig. 4b). ACR was associated with a stronger upregulation of T cell activation and interferon-inducible genes across multiple immune cell types.

Given the pivotal role of T cells in ACR, proliferating T cells displayed coordinated upregulation of lymphoid homing and trafficking genes (*CCR7*^29^, *SELL*^30^ and *CXCR3*^31,32^), consistent with enhanced recruitment of activated clones within the cardiac allograft. *CXCR3* expression in T cells has been linked with T cell-mediated graft injury previously^31,33^. In addition, costimulatory circuits were also upregulated in ACR (*CD27*^34^, *CD40*-*CD40L*^35,36^ and *SLAMF1*^37^), which collectively amplify T cell survival, cytokine production and dendritic cell licensing. In parallel, we observed increased expression of both effector/stress-adaptation genes (*GZMK*^38^, *ICAM1*^39^ and *FOSB*^40^) and exhaustion/checkpoint genes (*TOX*^41^ and *LAG3*^42^), a pattern typical of robust antigen-driven expansion under inflammatory constraint. Notably, negative regulators of T cell activation (*CD5*^43^) were also upregulated in ACR, suggesting feedback control in highly stimulated clones. Collectively, these data support an ACR-skewed state of robust T cell proliferation and tissue trafficking tempered by checkpoint induction rather than a purely ‘exhausted’ phenotype. Consistent with a dynamic role in acute inflammatory responses in immunologic injury, within T_reg_ cells we observed increased expression of *IL2RA*, which may serve as a ‘sink’ for locally produced IL-2 to curb inflammation^44^.

Within the innate immune compartment, cDC1 cells in ACR demonstrated upregulation of genes associated with enhanced antigen presentation and T cell priming capacity (for example, *CD80*^45^ and *FCGR1A*^46^), with additional increases in *CD68* and inflammatory mediators (for example, *IL1B* and *PRDM1*). Similarly, macrophages in ACR showed upregulation of interferon-responsive genes (for example, *IRF8*^47^) and antigen processing (*PSMB8*^48^). By contrast, mast cells upregulated genes associated with antigen processing and inflammatory crosstalk in ACR (for example, *CTSS*^49^, *ICAM1*^39^ and *SERPINB9*^50^) while in AMR, they showed relatively higher expression of canonical allergic/IgE-axis and prostaglandin D2 biosynthesis components (*MS4A2*^51^ and *HPGDS*^52^).

We also observed broad differences in other cell types between ACR and AMR. We identified pericyte-specific upregulation of complement-related genes^53^ (for example, *C4A*, *C4B* and *C7*) and inflammatory genes (*PLA2G2A*^54^) in ACR, potentially linked to loss of vascular integrity. Gene set enrichment analysis (GSEA) using hallmark gene sets identified that upregulated genes in ACR are significantly enriched (FDR <0.10) for IFNγ signaling in CD8^+^ T cells and macrophages, complement pathways in B cells and activated endothelial cells, and TNF/NFKβ signaling in cDC2 cells. (Fig. 4c and Supplementary Table 4). These pathways of inflammation, pro-coagulation and loss of vascular integrity are implicated in long-term CAV development, linking transcriptional changes during ACR not clearly captured by histology to later mechanisms of graft failure.

### DEGs between ACR and AMR are not fully recapitulated in bulk studies of EMBs

To understand whether DEGs identified in our study between ACR and AMR can also be identified in ‘bulk’ studies, we compared our findings with a publicly available EMB microarray dataset (GSE150059 (ref. ^55^)). We selected this dataset based on clear rejection classification, reasonable sample size and availability of annotated metadata. Of the 70 unique DEGs identified in our study, 65 were tested in the EMB microarray dataset. Comparing 76 patients with ACR and 179 patients with AMR, 34 shared genes were significantly differentially expressed between the conditions (Fig. 4d). When we compared the log_2_ fold change (FC) estimates across the two studies, we found the effects to be largely concordant with 53 of the 65 genes showing the same direction of effect (sign test *P* = 2.79 × 10^−7^)—validating many of the signals we see in our study. Nevertheless, 12 genes out of 65 showed discordant directionality. While these data support our findings, they also demonstrate how bulk approaches do not fully recapitulate what single-cell and spatial studies capture and may be less informative for precision subphenotyping of allograft rejection. One confounding factor in bulk studies of EMBs is that, due to small biopsy size, histological confirmation of rejection within a research biopsy may not be feasible. As rejection is often patchy, this presents a limitation of solely using bulk approaches on EMBs.

### Spatial transcriptomics reveals differences in transcriptional states between resolution and persistence of acute cellular rejection

Some patients experience ongoing rejection despite antirejection therapy. To subphenotype this unique state, we examined cell-specific gene expression differences in those who had resolution of ACR following antirejection therapies (responders) and those who did not (nonresponders). We prioritized ACR, given its higher frequency following HT and nonspecificity of immunomodulatory antirejection therapy (for example, corticosteroids and thymoglobulin)^6,11,12^. Of 32 individuals with ACR, 15 had resolution with antirejection therapies (17 did not). Among responders, we identified eight unique DEGs across five cell types (FDR <0.05; Supplementary Table 5). Treatment-associated decreases were seen in *CD8*^+^ T cells (*IRF8*^56^, *LYZ*^57^ and *LST1*^58^) and *BMX*^+^ activated endothelium (*VCAN*^59^), consistent with a dampening of inflammatory programs and endothelial remodeling after therapy. Among nonresponders (individuals with ongoing rejection), we did not observe significant changes in gene expression patterns despite antirejection therapy (Supplementary Table 6).

This led us to study pretherapy biopsies (all demonstrating rejection) to identify potential differences in the ‘baseline’ gene expression profile that may explain this difference. When comparing rejecting biopsies from responders versus nonresponders before antirejection therapy, we identified 23 significant gene–cell type pairs representing 21 unique DEGs (all FDR <0.05) across 6 cell types (Fig. 4e and Supplementary Table 7). The majority were in *CD8*^+^ T cells (ten genes), cardiomyocytes (six genes), and macrophages (three genes; Fig. 4f). Surprisingly, all significant genes had higher expression in ACR that was nonresponsive to therapy.

Nonresponders exhibited heightened baseline expression of T cell activation and tissue remodeling genes across several cell types. In *CD8*^+^ T cells, nonresponders had significantly higher expression of multiple T cell receptor signaling and activation markers (*CD3E*, *CD247*^60,61^, *CD28*^62^ and *CD69*^63^) along with those associated with memory T cell responses (*IL7R*^64^ and *GATA3*^65,66^). Macrophages showed higher expression of *MRC1*^67^, associated with the M2 phenotype and tissue injury responses. Furthermore, genes associated with extracellular matrix deposition and fibroblast activity were elevated in nonresponders. There was upregulation of *ASPN*^68^ in pericytes and *DCN*^69^ in cardiomyocytes and endothelial cells, both linked to remodeling. Taken together, these findings depict nonresponders exhibiting a more aggressive inflammatory profile at baseline compared with responders, aligning with the concept that higher initial immune activation and tissue injury burden may predict resistance to standard therapies (for example, steroid-refractory ACR). By contrast, GSEA of the same ranked comparisons revealed enrichment of pathways related to inflammation, TNF signaling and IL6–JAK–STAT3 signaling in vascular and stromal compartments in responders (Supplementary Table 8). This apparent divergence may reflect that, while nonresponders exhibit maladaptive T cell hyperactivation and tissue remodeling programs, responders may mount a more coordinated and pathway-level inflammatory response that remains susceptible to immunomodulation. Taken together, these data support the hypothesis that substantial molecular differences exist within ACR despite similar histopathological findings and that baseline differences in immune activation and stromal priming may help to explain divergent responses to immunomodulatory therapies^70–72^.

### Distinct cell-specific gene profiles are associated with CAV

**Fig. 5:**
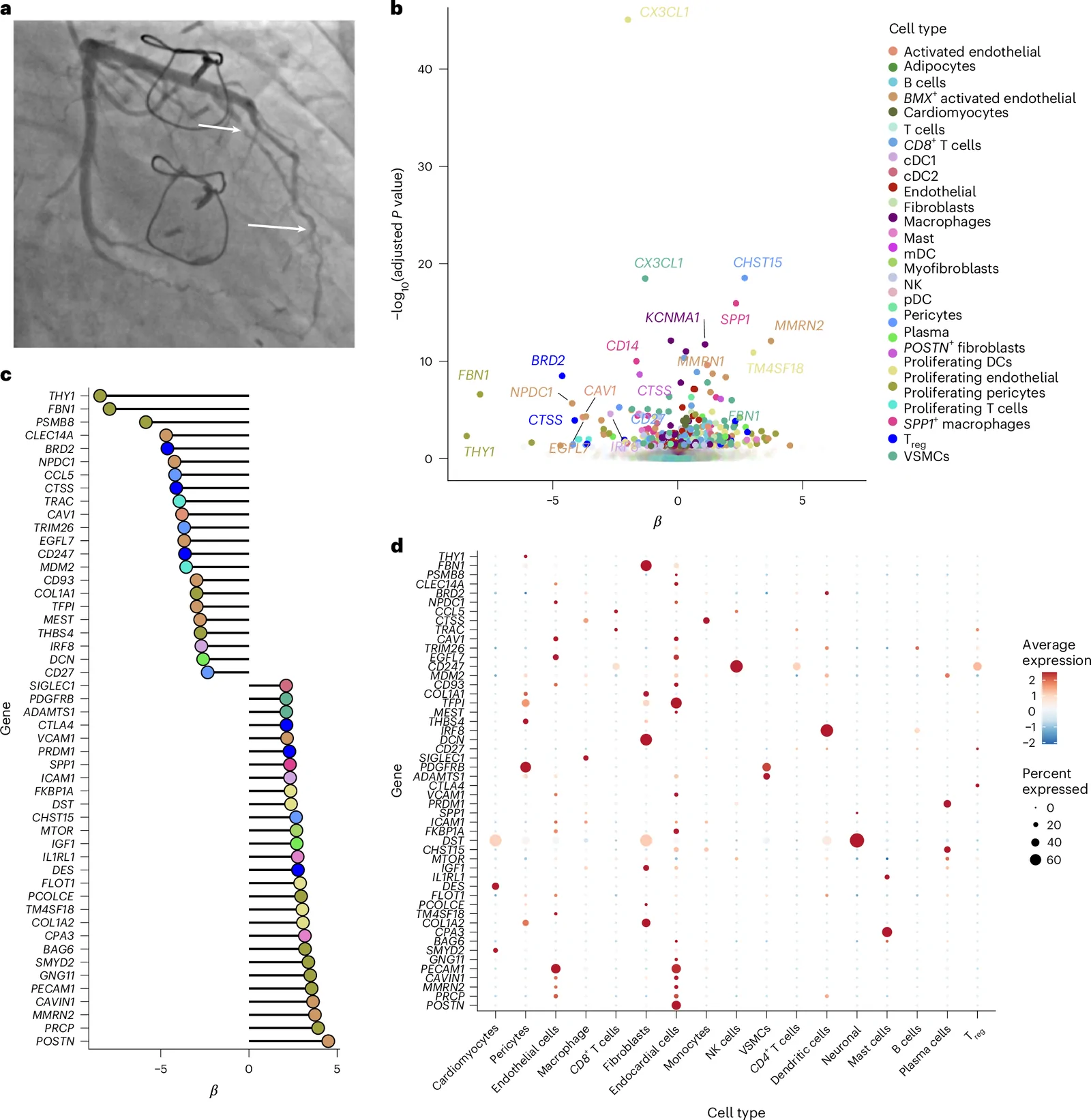
Cell-type-resolved expression profiles during acute rejection and following antirejection therapiesare associated with CAV. **a**, Coronary angiogram of a patient included in this study with moderate CAV (CAV grade 2). White arrows point to diseased portions of the left anterior descending artery and a diagonal branch. **b**, Association between cell-specific gene expression across biopsies spanning all three types of rejection and following immunomodulatory therapies. The *x* axis represents the *β* from the GLMM, while the *y* axis represents the −log_10_(*P* value). The color of each dot represents its corresponding cell type. Transparency reflects adjusted *P* values, with transparent dots representing those with adjusted *P* values ≥0.05. Associations were estimated using a GLMM (Gaussian family) with a patient-level random intercept, and significance was based on a two-sided test; *P* values were adjusted for multiple comparisons using the Benjamini–Hochberg FDR. Supplementary Tables 9–13 contain the full results of the GLMM models across all biopsies. **c**, The top 50 unique genes (arranged by absolute value of their *β* in the GLMM models) and cell types within those top betas are depicted. **d**, Dot plot of the same top 50 unique genes from **c** showing expression in a single-nucleus RNA-seq dataset consisting of right ventricular cardiac biopsies from patients with end-stage CAV requiring re-do HT.

To inform risk stratification for CAV, we related gene expression patterns during rejection and following therapy with CAV (Fig. 5a–c and Supplementary Tables 9–13). Across all biopsies, we identified 323 significant gene–cell type pairs representing 198 unique genes across 26 cell types. During ACR, we identified 59 unique genes in 12 cell types associated with CAV across pathways of angiogenesis (*ADGRL4*^73^ in vascular smooth muscle cells (VSMCs)), vascular inflammation (*CX3CL1*^74^ in VSMCs; the BET genes (for example, *BRD2*, *FGL2*^75^, *MEF2C*^76^), the multimerins^77,78^, *PLA1A*^79^ and *VCAM1*^80^ in *BMX*^+^ activated endothelial cells) and proliferation (*FAS*^81–83^ in VSMCs), and pro-coagulation (*CD40*^84^ in *BMX*^+^ activated endothelial cells). These pathways—activated during ACR—have been implicated in CAV. Several genes have been previously identified in microarray studies of biopsies during acute rejection^79,85^. Here, we demonstrate that these genes are expressed in a cell-specific fashion during acute rejection and are associated with CAV.

Following antirejection therapies in ACR, which are expected to attenuate myocardial inflammation, we tested for “residual” cell-specific inflammation that may be associated with CAV. We identified 45 unique significant genes across 16 cell types associated with CAV. Only nine genes overlapped with those identified during ACR, suggesting evolving molecular processes despite histological resolution. The largest number of associations were in cell types implicated in CAV: those of the microvasculature (pericytes), supporting cells (fibroblasts) and antibody-producing cells (B cells and plasma cells). The strongest associations with CAV included *COL1A2* in proliferating endothelial cells and *MTOR*, *FLOT1* and *DES* expression in myofibroblasts. Currently, mTOR inhibitors are part of a limited set of therapies in the armamentarium against CAV. Our findings of early *MTOR* expression following rejection treatment specifically in myofibroblasts, previously shown to drive fibrosis and vasculitis^86–88^, may offer an opportunity for early interruption of CAV pathogenesis following rejection. GSEA identified enrichment of pathways involved in endothelial-to-mesenchymal transition among cell types of the vasculature (pericytes, VSMCs and fibroblasts; FDR <0.10; Supplementary Table 14), which we have previously identified in the myocardium of individuals with severe CAV^28^. These results show that, despite antirejection therapies, residual inflammatory risk (unmeasured by histology alone) persists and is associated with CAV.

In AMR, 56 genes across 16 cell types were associated with CAV. Significant gene associations predominantly spanned microvasculature and supporting cell types (VSMCs, proliferating pericytes, endothelial and activated endothelial cells). These include genes associated with vascular integrity and angiogenesis (*FAS*^83,89^ in activated endothelial cells; *PECAM1*^90^, *SMYD2*^91^ and *PCOLCE*^92^ in proliferating pericytes; *FBN1*^93,94^, *ACTG2*^95^ and *OGN*^96^ in VSMCs) and inflammation (*IRF8*^97^, *ICAM1*^98^ and *IL6R*^99^ in cDC1; *ITGAM*^100^ and *SLAMF7*^101^ in NK cells; *CD69*^63^ in CD8^+^ T cells), among others. Following therapies, 91 genes across 17 cell types remained associated with CAV. The overwhelming majority of gene associations following AMR treatment remained concentrated in cardiomyocytes, microvasculature, *CD8*^+^ T cells, and macrophages. These included genes related to endothelial integrity and inflammation (*PECAM1*^90^, *CD93*^102^, *FBN1*^94,103^, *TCF4*^104^, *VCAN*^22^ and *MTOR*^86–88^ across subsets of endothelial cells) and fibrosis (*POSTN*^105^ in endothelial cells; *PDGFRA*^106^ and *PDGFRB*^107^ in fibroblasts). As expected, persistent inflammation across a variety of immune cells was prominently associated with CAV. Similar gene associations between mixed rejection and CAV were also identified, including 13 and 9 shared genes with AMR and ACR, respectively.

### Spatial transcriptomics identifies cell-specific genes altered during and following acute rejection that are dysregulated in severe CAV

**Extended Data Fig. 10:**
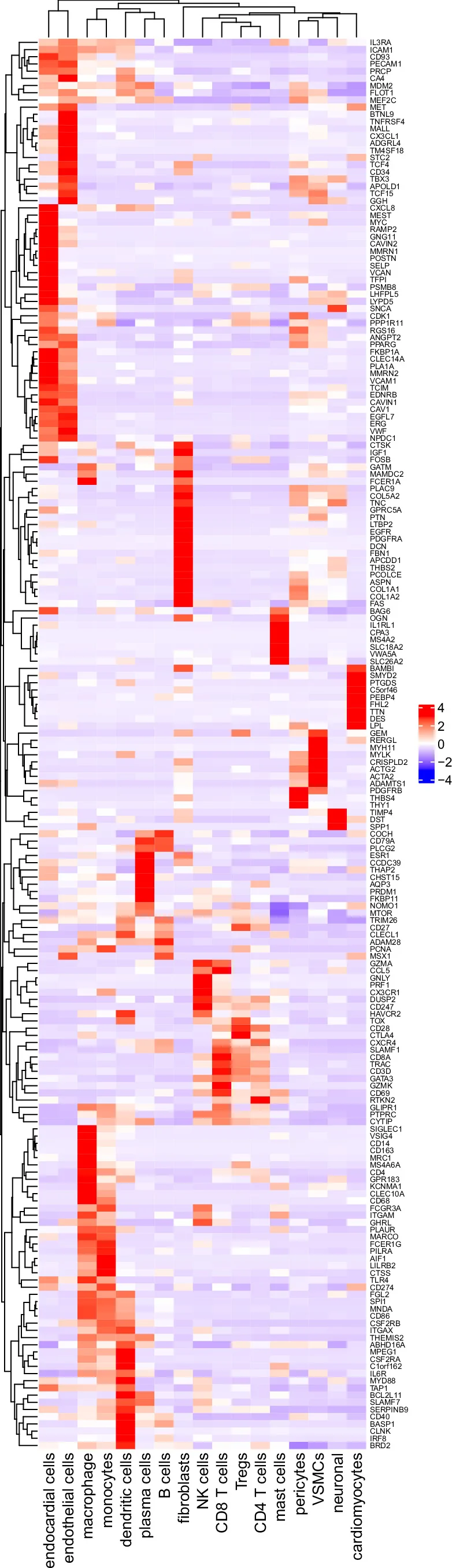
Cell-specific expression profiles of CAV-associated genes in end-stage coronary allograft vasculopathy. Heatmap of cell-specific gene expression patterns in a published single-nucleus RNA-sequencing dataset of end-stage CAV. Genes visualized are those significantly associated with CAV in this Xenium study.

To evaluate whether CAV-associated genes were expressed in a similar cell-specific fashion in the myocardium following overt CAV development, we turned to our previously published single-nucleus RNA-seq dataset that sampled left and right ventricular tissue from the human heart in CAV^28^. We observed gene expression in a similar cell-specific fashion (for example, high expression of *PECAM1*, *VCAM1*, *ICAM1*, *CD93*, *POSTN* and so on in endothelial and endocardial cells; *MYD88* in dendritic cells and monocytes; *IL6R*, *ITGAX* and *IRF8* across multiple immune cell types; Fig. 5d and Extended Data Fig. 10) in severe CAV. These data suggest that cell-specific gene expression during rejection and following treatment—particularly genes involved in residual inflammation, angiogenesis/vascular integrity and antifibrotic responses—are significantly expressed in a similar cell-specific fashion in end-stage CAV myocardium, transcriptionally linking ‘early’ immunological phenomena to long-term CAV.

## Discussion

Tissue-based molecular phenotyping is *sine qua non* of modern precision approaches across a variety of disease states^108^. Penetrance of tissue genomic methods to subphenotype disease has been especially fruitful in diseases where cell-level heterogeneity is the rule (for example, oncology^108–110^). While most chronic diseases do not lend themselves to tissue analyses, solid organ transplantation represents a unique setting where tissue may be routinely obtained for rejection surveillance and to guide therapy^7,10^. Unfortunately, variability in histologic interpretation challenges precise application of immunomodulatory therapy (and downstream immunosuppression-related chronic clinical consequences^111^). Studies that directly examine tissue molecular states and dynamic changes with therapy across different modes of rejection are urgently needed to translate genomic advances to this space.

Here, we answer this call by conducting spatial transcriptomics in 62 longitudinal adult and pediatric cardiac allograft biopsies to characterize acute rejection following HT, dynamic responses to immunomodulatory therapies, and link cell-specific gene expression patterns to the development of chronic rejection (that is, CAV). Our principal findings show (1) a diverse array of cell types are involved in acute rejection and response to immunomodulation. (2) There was substantial molecular heterogeneity both across rejection types and within the same rejection grades, masked by histology alone. Differences in gene expression patterns between AMR and ACR were only partially captured in an external dataset. (3) Baseline rejection biopsies are composed of distinct transcriptomic profiles between patients who go on to respond to immunomodulatory therapies versus those who do not. (4) Cell-specific gene expression patterns both during rejection and following immunomodulatory therapies are associated with CAV. These genes showed similar cell-specific expression patterns in an external single-nucleus RNA-seq dataset of myocardium obtained from patients with end-stage CAV. Our findings link molecular heterogeneity with clinical heterogeneity and advance our knowledge of how acute rejection may mediate CAV. These are highly relevant clinical processes that are incompletely understood at this time. Furthermore, this unique dataset serves as a public resource to guide future studies on acute rejection diagnosis, therapeutic response prediction and risk stratification for CAV development.

The current gold standard for rejection diagnosis remains histology supplemented with limited immunofluorescence or immunohistochemistry^7,10^. However, interpathologist variability in the grading of rejection is substantial^9^. More importantly, histology alone has failed to explain heterogeneity in clinical presentation, whether individuals will respond appropriately to antirejection therapies, and in risk stratifying for future CAV development. Using principal component analysis (PCA) and hierarchical clustering, we show that substantial molecular heterogeneity is present both across different types of rejection (ACR, AMR and mixed) and within the same grades of histologic rejection (for example, during grade 2 R ACR). This latter finding is particularly important clinically, as it may help explain why a spectrum of clinical presentations—spanning complete lack of symptoms to florid cardiogenic shock—exists within the same histologic grade of rejection and why some individuals have resolution of rejection with minimal intervention (for example, outpatient augmentation of oral prednisone) while others require broadly-targeting T or B cell-depleting therapies (for example, thymoglobulin, intravenous immunoglobulin, plasmapheresis and rituximab).

Furthermore, while multiple observational studies have shown that acute rejection is a major risk factor for CAV development^14,112^, it remains incompletely understood as to how transient episodes of acute rejection are linked to CAV. While animal models have suggested that IFNγ signaling and interactions between both lymphoid and myeloid cells with coronary endothelial cells and VSMCs may play important roles^113,114^, direct clinical translation has been limited by lack of clinical levels of immunosuppression. Our results in human cardiac tissue surrounding acute rejection highlight a number of cell-specific genes associated with CAV. Notably, the majority of significantly-associated genes are in cells of the vasculature (endothelial cells, *BMX*^+^ activated endothelial cells and pericytes) and supporting cells (fibroblasts and VSMCs) and span across pathways of angiogenesis (*ADGRL4*^115^ in fibroblasts), vascular inflammation and proliferation (*CX3CL1*^74^ in VSMCs and proliferating endothelial cells; *ITGAM*^93^ in NK cells; the BET genes (for example, *BRD2*), *FGL2*^75^, *MEF2C*^76^, the multimerins^77,78^, *PLA1A*^79^, and *VCAM1*^80^ in *BMX*^+^ activated endothelial cells; *TNFRSF4*^116^ in NK cells; *FAS*^81–83^ in VSMCs and endothelial cells), and promotion of coagulation/complement fixation. These microvasculature-directed transcriptomic changes suggest the potential for spatial molecular phenotyping approaches in risk stratifying individuals for future CAV. Importantly, CAV-associated genes that we identify in our study (‘early’ in disease course) are expressed in a similar cell-specific fashion in myocardium from patients with end-stage CAV requiring re-do HT.

As spatial technologies evolve to capture the whole transcriptome at subcellular resolution, prioritizing study of longitudinal EMBs may further uncover how cell-specific fates diverge between individuals with healthy allografts and those who develop rejection or challenges to long-term graft survival (for example, CAV). The few studies performed so far have largely prioritized ‘bulk’ approaches (mostly microarray studies) to anchor molecular patterns to pathologist-given histologic grade^55,79,117–122^. These studies have been challenged by lack of cellular/subcellular resolution (‘washing out’ cell-specific gene expression patterns) and inadequate resolution of the coronary microvasculature only accessible in modern single-cell and spatial techniques (relevant to CAV), among other limitations. Data generated through approaches such as ours (for example, spatial/single-cell transcriptomics) during a variety of therapies and long-term immunomodulatory strategies (for example, mTOR inhibition) may substantially inform early biomarker and drug discovery to meaningfully prolong allograft survival in the modern era^123,124^.

Key strengths of this study include a large sample size spanning adult and pediatric HT, longitudinal human cardiac biopsies from the same patients, and modern spatial transcriptomics at subcellular resolution to interrogate cell-specific gene expression patterns. Furthermore, cardiac biopsy samples in our study were obtained from individuals without end-stage graft dysfunction, which carries a distinctive transcriptomic profile that may not reflect early, modifiable mechanisms^21,105,125–128^. Evaluating non-end-stage allografts allows us to directly map the dynamic immune–cardiac interactions during rejection and treatment, providing a clearer biological baseline uncoupled from the effects of advanced disease. Nevertheless, our study does have important limitations. Histologic grade, time from transplant, and treatment type are potential confounders that could influence both the trajectory of rejection and gene expression profiles. While we obtained tissue samples during standard clinical care and with real-world constraints, our findings should be interpreted in the context of several limitations inherent to current spatial transcriptomic technologies and tissue sampling methods. First, 2D spatial transcriptomic sections do not fully recapitulate the three-dimensional architecture of the myocardium and, thus, may not accurately reflect true cell type proportions across the entire tissue volume. Second, technical limitations in cell segmentation and annotation—known limitations of 2D spatial transcriptomics—can result in a subset of cells being unlabeled or misclassified, potentially introducing bias into downstream analyses. Furthermore, because rejection in EMBs is often patchy and can co-exist with fibrosis/scarring, focal TMA sampling cannot fully capture all histologic heterogeneity across the biopsy set. However, this same patchiness provides an opportunity to study local molecular states within pathologist-selected regions of interest. While comparisons to external bulk- and scRNA-seq datasets provide useful context, these references are themselves subject to dissociation-related artifacts and batch effects, limiting their ability to serve as a definitive ground truth for in situ cellular composition. To mitigate these concerns and ensure rigor and reproducibility, we provide high-resolution H&E images for all analyzed sections and report the proportion of segmented but unlabeled cells, allowing readers to directly assess tissue quality and analytic coverage. Despite sampling biases inherent in modern EMB techniques, invasive biopsy remains the current gold standard of clinical care after HT. To limit variability, we used a single, experienced cardiac pathologist to review whole-slide gradations of rejection, as well as TMA-specific gradation. Despite our sample size and longitudinal sampling, additional gene discovery in larger samples is warranted to expand mechanistic implication. In addition to careful tissue processing and conservative analytic approaches, larger cohorts will be important to mitigate local sampling variability and more fully capture the biological heterogeneity inherent to clinically collected EMBs. Furthermore, our approach (Xenium) uses a targeted probe set informed by our prior experience and published literature. There may be potential underrepresentation of certain cell types due to the use of a targeted probe panel. Finally, we only compared our results with one published bulk microarray dataset, which we chose specifically due to its clear rejection classification in available metadata. However, differences in platform (subcellular spatial transcriptomics versus bulk TMA) and technical variations in experimental protocols may confound the results. We believe that larger validation studies, ideally across independent and multicenter cohorts and incorporating dedicated orthogonal RNA- or protein-based assays, will be needed to fully establish the magnitude, reproducibility and clinical relevance of these phenotype-specific molecular differences. Important in interpreting the results of our study, our goal was to assess concordance between spatially derived cell-specific gene signatures and previously reported bulk-level rejection signals in an orthogonal context. Nevertheless, our findings are biologically plausible with replication in single nuclear data. We anticipate that, as technologies advance in conduct and economy, unbiased whole transcriptomics at subcellular resolution will substantially add to our findings, including allowing us to realize the potential for these approaches in forecasting rejection grade and resolution.

In conclusion, our study establishes significant molecular heterogeneity across rejection types and within the same rejection grades unapparent by histology alone in adult and pediatric HT. Furthermore, we identify distinct cell-specific gene expression patterns, only partially recapitulated in bulk studies, across dynamic responses to therapy using longitudinal cardiac biopsies. Finally, we link cell-specific gene expression to highly-relevant clinical outcomes (that is, chronic rejection (CAV)) and show similar cell-specific gene expression in end-stage CAV. Together, our findings elucidate cellular dynamics in cardiac allograft rejection and provide a valuable resource for the larger transplant scientific community. We anticipate this work to be a step toward precision approaches in solid organ transplantation.

## Methods

### Human samples and study approval

This study was conducted with the approval of the Vanderbilt University Medical Center institutional review board (IRB #200551). The overall study design is depicted in Fig. 1a. Formalin-fixed paraffin-embedded (FFPE) EMB samples were obtained from adult and pediatric HT recipients at the Vanderbilt University Medical Center undergoing routine surveillance or for-cause endomyocardial biopsies between December 2017 and January 2023. Rejection grading was performed by clinical pathologists and confirmed by pathological review by an experienced cardiovascular transplant pathologist (R.D.H.) at both the whole-slide and TMA levels to limit spatial variability. Only biopsies exhibiting clinically significant acute rejection were selected, along with corresponding posttreatment biopsies. Rejection phenotypes were defined using accepted ISHLT criteria^7,8,10^. ACR was defined as grade ≥2R, which is characterized histologically by two or more foci of mononuclear inflammatory infiltrates with associated myocyte damage. AMR was defined as pAMR1-i or pAMR2/3, based on histologic and immunopathologic criteria. pAMR1-h indicates histologic evidence of endothelial injury (for example, capillary endothelial swelling) without C4d deposition, while pAMR1-i involves C4d deposition in ≥50% of capillaries. pAMR2 combines both histologic and immunologic findings while pAMR3 denotes severe AMR with concomitant hemodynamic compromise. Biopsies demonstrating both ACR and AMR features on the same sample were classified as mixed rejection.

### TMA construction

To minimize batch effect and improve run efficiency, multiple samples were placed on a single Xenium slide (available sample positioning area measuring 10.45 mm × 22.45 mm) using a TMA. Slides of each FFPE EMB block were H&E stained for clinical diagnosis and were evaluated by an experienced cardiovascular transplant pathologist (R.D.H.), who selected two regions of interest per block, inclusive of the area of rejection. In EMBs without clinical rejection, two normal regions were selected. TMA blocks were constructed to target two 0.6-mm cores per FFPE block, and a total of 233 cores underwent sectioning at 5 µm thickness before being placed onto two Xenium slides. After drying overnight, the slides were placed in a sealed desiccator and stored at room temperature for ≤7 days.

### Xenium workflow

This workflow uses targeted gene panels that can be custom designed. A total of 477 unique genes were included in this study. Of these, 377 were in a predesigned panel (Human Multi-Tissue and Cancer), which include markers of a large panel of immune cells as well as cells comprising the human myocardium. An additional 100 custom genes were selected, informed by published single-nucleus RNA-seq^28^, as well as bulk RNA-seq and microarray studies^55,79,119–122^. The full list of genes is presented in Supplementary Table 15.

As part of sample preparation, all workstations and equipment were cleaned using RNAse Away (RPI 147002) and 70% isopropanol. Following placement on Xenium slides, tissue samples were deparaffinized and decrosslinked to access mRNA. Hybridization using the gene panel probes occurred for 18 h at 50 °C, followed by washing of unbound probes. Next, probes were ligated at 37 °C for 2 h, followed by rolling circle amplification for 2 h at 30 °C. The slides were then washed and then stained with the 10x Genomics commercial kit for improved cell segmentation. Background fluorescence (present in a number of tissues, including heart) was quenched chemically. Additional washes with PBS and PBS-T were undertaken, nuclei were stained using DAPI, and slides were loaded onto the Xenium Analyzer instrument version 2.0.1.0.

Following loading of consumable reagents and the two Xenium slides, the Xenium Analyzer instrument decodes subcellular localization of mRNA targets in a fully automated fashion. After binding of fluorescently labeled oligos, samples underwent multiple cycles of hybridization, followed by acquisition of images and probe stripping are undertaken with images taken at 4,240 × 2,960 pixel fields of view. As each gene in the panel has a unique fluorescence pattern across image channels, focal areas of intensity were decoded and labeled according to the gene ID. All image fields of view and transcripts were stitched together using the DAPI-stained image as the anchor, followed by quality control for each detected transcript (instrument software version v1.1.2.4 and xenium-v1.1.0.2). Following the run on the Xenium Analyzer instrument, the chemical quencher was removed and slides underwent H&E staining. Histology images were taken using the 20x Leica Biosystems Aperio CS2 instrument (Supplementary Figs. 6 and 7). Cell segmentation was performed using the 10x Xenium software. All analyses were performed at the cell level.

The Xenium images and H&E images were integrated using ImageJ (v 2.14.0) based on identifiable landmarks on both images. Registered images were manually reviewed for any potential artifacts resulting from this process. In the Xenium Explorer 3.0.0, we used the lasso function to obtain cell IDs corresponding to each visible core/punch. Of the 233 targeted cores, 195 punches were lassoed and used in downstream analyses. The output files include coordinates in 2D space, corresponding gene, assigned cell and quality metrics. Using this output, low-quality transcripts (quality value (qv) <20) and transcripts that corresponded to blank probes were removed. Subsequently, Seurat (v5.0.1)^129^ was used, and cell-level count data were loaded into RStudio (R 4.3.0-4 and 4.4.0-3)^130^ using Seurat’s LoadXenium function with default settings. Cells outside of lassoed regions were excluded and cells with 0 counts were removed from subsequent analyses, leaving a total of 189,109 cells. Cells were retained based on ≥12 transcripts corresponding to ≥10 unique genes, percentage of high-quality transcripts corresponding to blank probes ≤5, and nucleus area ≥6 μm and ≤70 µm. A total of 164,165 cells remained after filtering.

Dimensionality reduction and clustering were performed using a Docker container with RAPIDS (v21.8.1)^131^, which implements Scanpy (v1.8.1)^132^ in Python (v3.10.13)^133^, while Seurat (v5.0.1) was used for downstream analyses and visualizations. RNA counts matrices underwent log1p normalization. PCA was used for dimensionality reduction. A nearest-neighbor distance matrix and a neighborhood graph were computed using 20 PCs and 20 nearest neighbors and were used to generate a uniform manifold approximation and projection (UMAP). Clustering was performed using the Leiden algorithm. Cell types were annotated using a similar approach as our prior work^134^, where first-pass annotations were implemented to define the main lineages and a second pass was performed for fine-level granular annotations. To identify cell types, we assessed expression of canonical marker genes and top markers using Presto (v1.0.0)^135^ wilcoxauc and top_markers functions (Extended Data Fig. 1). This resulted in 28 cell types including 5 endothelial, 15 immune and 8 mesenchymal. After annotation, we excluded cells from one patient who was not related to this study from subsequent downstream analyses, leaving a total of 162,638 cells. Further metadata of TMAs are presented in Supplementary Table 16.

### Immunohistochemistry

Immunohistochemical stains for CD3, CD8 and CD68 were performed on 15 whole-slide EMBs distinct from those used for spatial transcriptomics. These included five nonrejecting EMBs and five each comprising ACR and AMR, respectively. H&E- and immunohistochemically stained sections were digitally imaged using Aperio AT2 scanners at 40× magnification. QuPath^136^ (v0.6.0) was used for image analyses. Immunohistochemical images for each case were assessed to determine the proportion of cells expressing each antibody using the positive cell detection feature. Default settings were used for the majority of cases, with minimal adjustments to attenuate large-scale inaccuracies. Quilty lesions, regions of harvest injury (ischemia–reperfusion injury that persists for weeks following HT), extraneous noncardiac tissue floaters and areas of stain precipitant were excluded manually. Results are reported as percentage of cells with cytoplasmic/membranous expression of the marker compared with total cells in the field. Comparisons between groups were conducted using the Wilcoxon signed-rank test. Each scanned slide was examined by a cardiac pathologist with dedicated expertise in transplant (A.N.P.) to ensure accuracy of cell quantification.

### Comparison with scRNA human heart atlas

To compare lineage-level cell type recovery in our data, we compared our results with the human Heart Cell Atlas v2 single-cell/nuclei RNA and ATAC-seq dataset^125^. We included all samples from the Heart Cell Atlas (HCA; 25 adult donors, 14 females and 11 males). Because the HCA dataset comprised unaffected donors and our dataset was from heart transplant patients, we separated our biopsies into two groups—biopsies without evidence of rejection (normal myocardium) and biopsies with rejection—when comparing lineage proportions rather than aggregating all cells for each patient. For the HCA data, the proportion of cells from each lineage was calculated for each donor, while for our data, the proportion of cells from each lineage was calculated for each biopsy.

### PCA

To assess the heterogeneity across acute rejection we performed PCA using the factoextra R package^137^. PCA was performed on matrices of cell type proportions and on pseudobulked expression for pretreatment samples. Cell type proportions were calculated for each biopsy. For the expression PCAs, the log1p-normalized data were averaged per gene per patient across all cell types and for each cell type separately.

### Transcriptional similarity analysis

To characterize molecular differences across ACR severity, we stratified pretreatment ACR biopsies by TMA-level histologic grade to compare transcriptional profiles from the sampled regions themselves. We then identified the closest cells in the transcriptional space within each cell type. We ran RunPCA() and FindNeighbors() in Seurat for all cells from the same biopsy grade and for each cell type separately (source cell type). Then, the nearest-neighbor graph used to select the cell with the most transcriptional similarity to the source cell. A Fisher’s exact test was then performed for each source biopsy grade, by grouping source cells of the same biopsy grade and not, and if the closest transcriptional neighbor was from the same biopsy grade as the source cell or not. *P* values were adjusted using the FDR correction method.

### Cell type proximity analyses

To assess physical proximity between cell types, we used the approach we previously established^134^. In brief, distance and angular direction were calculated between each cell and its neighboring cell within a 60-µm-radius circle. The probability of cell types being proximal to one another was assessed using a Fisher’s exact test by assigning cells to binarized proximal and nonproximal categories. *P* values were adjusted using the FDR correction method.

### Cell type proportion analyses

Cell type proportion differences were statistically assessed among AR types using the propeller method within the speckle R package^138^. Using the propeller default function, cell type proportions were calculated at the biopsy level for all pretreatment biopsies, proportions were transformed using the ‘logit’ method, and an analysis of variance was performed.

### Differential gene expression testing

We performed cell-type-specific differential expression analyses using linear models. For comparisons of pretreatment ACR versus pretreatment AMR, and pretreatment versus posttreatment biopsies among ACR patients with resolution, we aggregated expression at the patient level. For the pretreatment comparison of ACR responders versus nonresponders, we aggregated expression at the biopsy level and included TMA grade as a covariate in the linear model to account for differences in baseline histologic severity between groups (Supplementary Fig. 8). For each cell type, aggregated expression profiles (‘pseudobulked’ profiles) were generated by averaging log1p-normalized expression values for each gene within each patient or biopsy, as appropriate. We excluded cell types represented by fewer than three samples per group and retained genes detected in at least three aggregated samples. Because spatial transcriptomic data are susceptible to segmentation-related technical artifacts, including biologically implausible transcript-to-cell assignments, we applied an additional filtering step before differential expression testing. Specifically, we used an external human heart scRNA-seq atlas to identify genes that should be plausibly expressed within a corresponding cell type of the human heart. We grouped our cell types into the following categories: endothelial, mural, myeloid, fibroblast, lymphoid, cardiomyocyte, lymphatic endothelial, mast and adipocyte. For each gene in our panel, we analyzed that gene only in cell types where at least 1% of the cells expressed that gene from the corresponding group in the atlas. This filter was used to reduce the influence of likely segmentation artifacts rather than to infer true in situ cell proportions. GSEA was performed using the fgsea and msigdbr packages, prioritizing Hallmark gene sets.

### Generalized linear mixed models (GLMMs)

We performed a series of GLMMs to assess whether expression is associated with outcome (CAV grade) in ACR patients. GLMMs were performed using glmmTMB package in R (ref. ^139^). Patient age was added as a fixed effect, and patient ID was added as a random effect. We pseudobulked per biopsy by averaging the log1p-normalized data per gene and per biopsy for each cell type separately. Genes were removed from analysis if there were fewer than five samples with expression. *P* values were adjusted using FDR correction method. Gene–cell type pairs with convergence issues (either false convergence or function evaluation limit reached without convergence) were not included in the analysis. There were a total of 18 gene–cell type pairs across all GLMMs that had convergence issues. In addition, we used the same approach as with the linear models to remove genes based on their expression in the scRNA human heart atlas.

### Validation of DEG results with a publicly available microarray dataset

GeoQuery was used to download microarray data (GSE150059)^55^. Data were first evaluated for quality and gene distribution. Subsequently, only biopsies labeled ‘ABMR’ (representing AMR) and ‘TCMR’ (representing ACR) were included for downstream analyses. The limma pipeline (lmFit) was used for differential expression analyses. Multiple testing correction was performed by the Benjamini–Hochberg correction. Genes were considered significantly differentially expressed if the Benjamini–Hochberg-corrected *P* value <0.05 and absolute(log_2_FC) >0.58.

### Validation of CAV-associated gene expression in single-nucleus RNA-seq of end-stage CAV myocardium

Prior data from our group focused on single-nucleus RNA-seq of cardiac biopsies from end-stage CAV patients undergoing re-do transplant and non-CAV controls were utilized, with data processed as previously described^28^. As CAV tissue was sampled from both the right and left ventricles, we prioritized right-ventricle samples to maintain concordance with our current study (as EMBs are from the right ventricle). Normalized gene expression outputs were used for visualization.

### Statistics and reproducibility

All methodological approaches are outlined in detail within the relevant portions of the Article and in the Methods. Sample size for each analysis is reported alongside results. We did not perform sample size calculations before undertaking this study. This study did not include any randomization or blinding. To enhance rigor and reproducibility, all raw and processed data are shared as described in the ‘Data availability’ section, and code used for analyses is shared as described in the ‘Code availability’ section.

### Reporting summary

Further information on research design is available in the Nature Portfolio Reporting Summary linked to this article.

## Supplementary information

Supplementary Information Supplementary Figs. 1–8.

Reporting Summary

Supplementary Tables 1–16 This file contains Supplementary Tables 1–16.

Supplementary Data 1 This file contains source data for Supplementary Figs. 2, 4–6 and 10.

## Source data

Source Data Fig. 1 Statistical source data for Fig. 1d,e.

Source Data Fig. 2 Statistical source data for Fig. 2a,c.

Source Data Fig. 3 Statistical source data for Fig. 3b–d.

Source Data Extended Data Fig. 1 Statistical source data for Extended Data Fig. 1.

Source Data Extended Data Fig. 9 Statistical source data for Extended Data Fig. 9.

## Data Availability

Raw and processed data are deposited on GEO under accession GSE290577. These include high-resolution H&E and immunohistochemistry images (high-quality.svs files in GSE290577_IHC_raw_images.tar.gz).

## Code availability

Code relating to the analyses for this Article is available via GitHub at https://github.com/Banovich-Lab/Spatial_Heart_Rejection.
