# Supplementary material for: Dynamic cellular programs of human cardiac allograft rejection revealed by spatial transcriptomics
Source: Nat Cardiovasc Res. 2026 Aug 10;5(9):819–34. doi: 10.1038/s44161-026-00849-9 (PMC13561861; doi:10.1038/s44161-026-00849-9)
Supplement: Supplementary file 1 [file 44161_2026_849_MOESM1_ESM.pdf]

nature cardiovascular research

Supplementary information https://doi.org/10.1038/s44161-026-00849-9

Dynamic cellular programs of human cardiac allograft rejection revealed by spatial transcriptomics

In the format provided by the authors and unedited

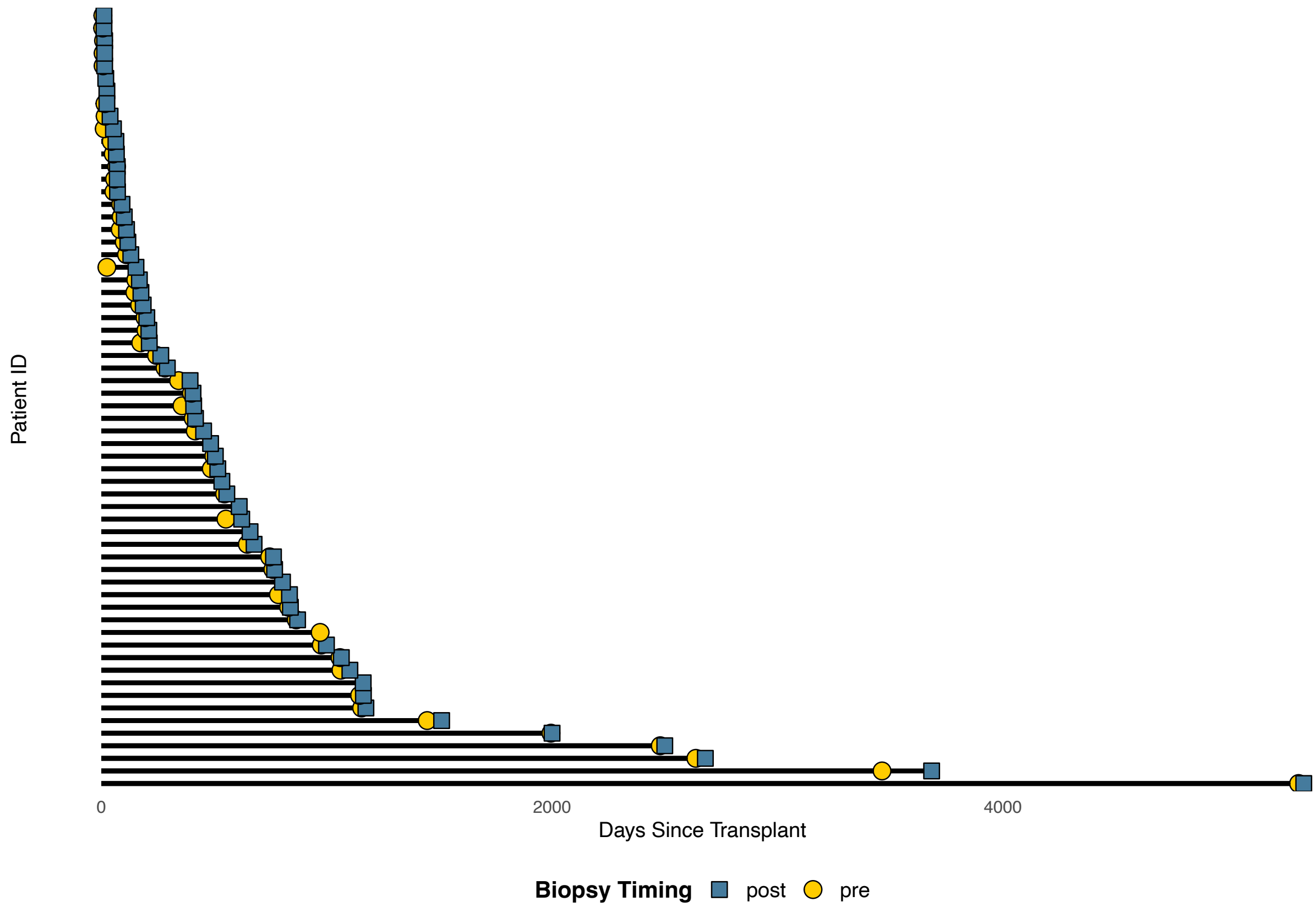

Supplementary Figure 1. Swimmer's plot of timing of EMBs included in this study after HT.

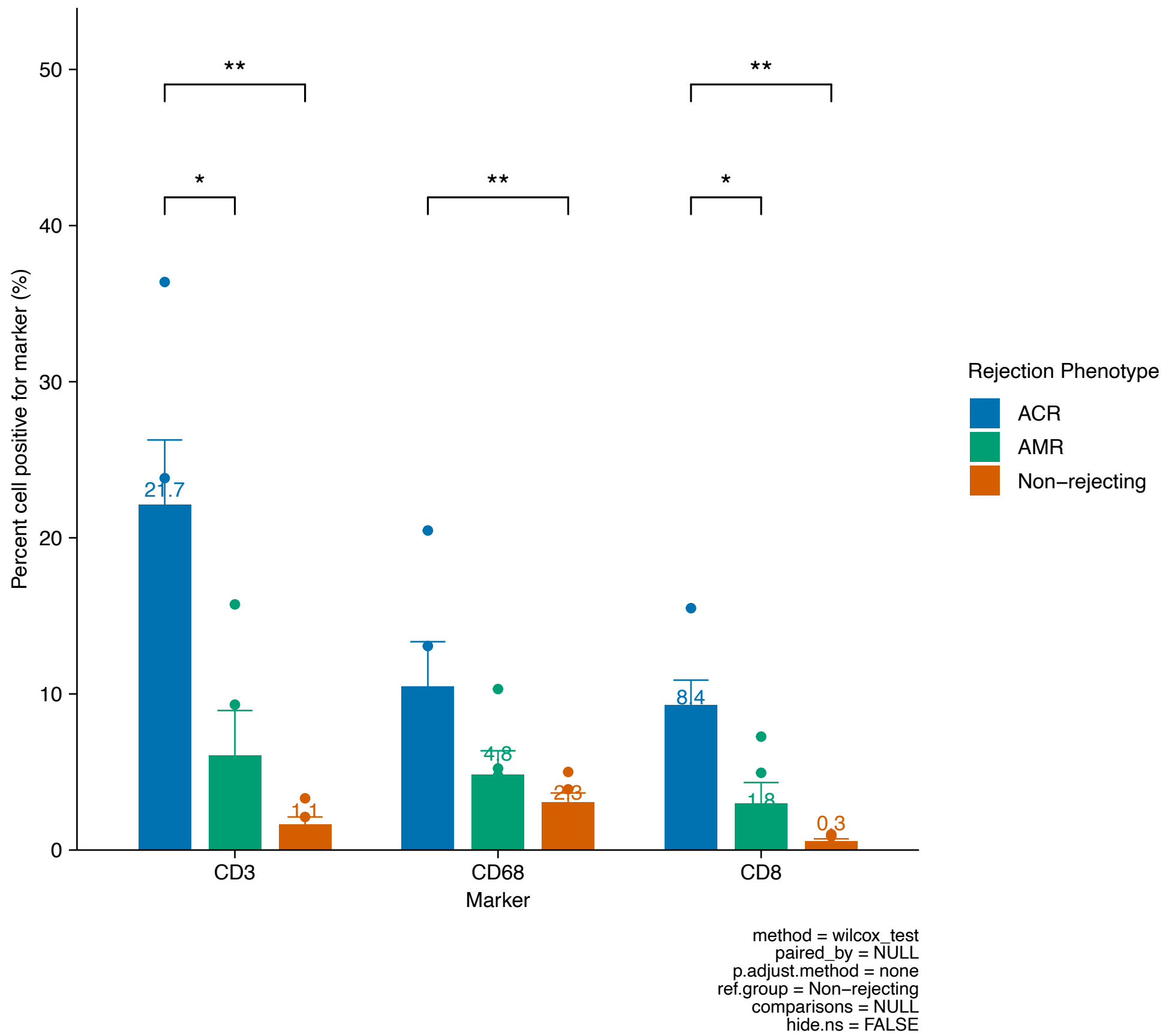

Supplementary Figure 2. Immunohistochemistry results using CD3, CD68, and CD8 between non-rejection, ACR, and AMR biopsies (N = 5 in each group). Whole EMBs were used. Regions containing Quilty lesions, ischemia-reperfusion (termed "harvest injury"), extraneous non-cardiac tissue floaters, and areas of stain precipitant were manually excluded following review by a cardiovascular transplant pathology. The results are reported as the percentage of cells

with expression of the marker compared with total cells in the field. Between-group comparisons were performed using the Wilcoxon signed-rank test. *P < 0.05; **P < 0.01.

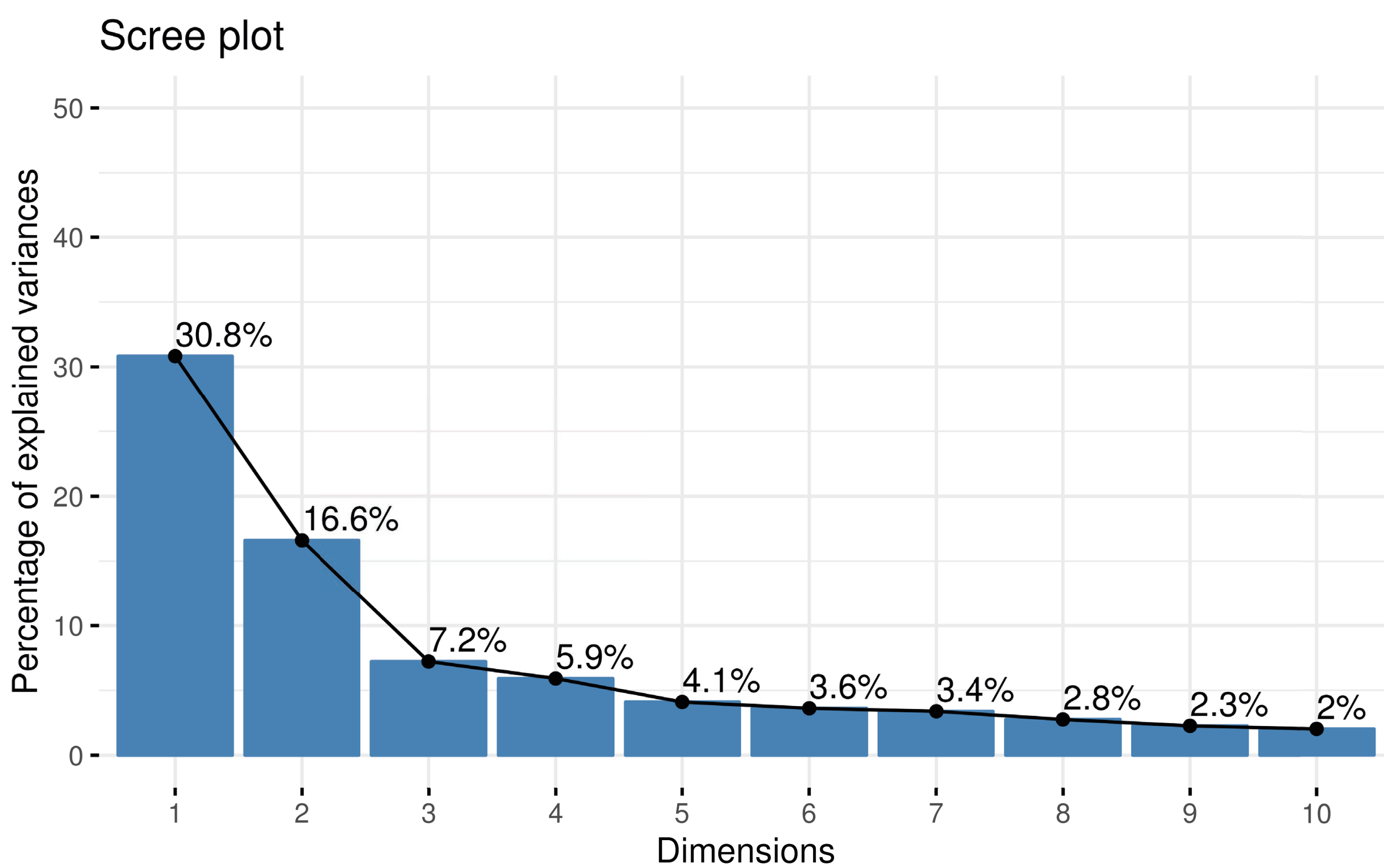

Supplementary Figure 3. Scree plot demonstrating percent of variance explained by each principal component (x-axis).

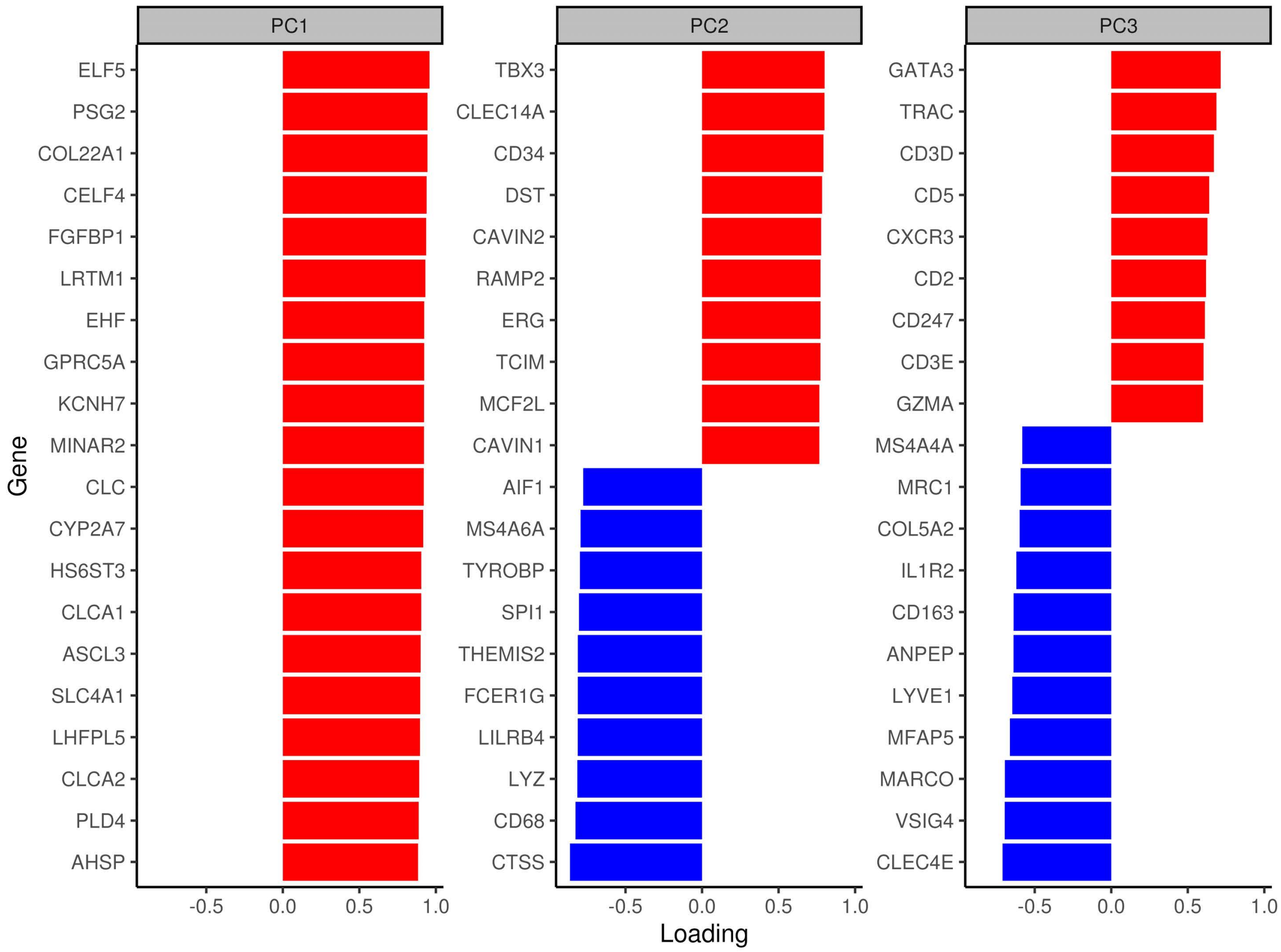

Supplementary Figure 4. Top 20 gene loadings for each of the first 3 principal components.

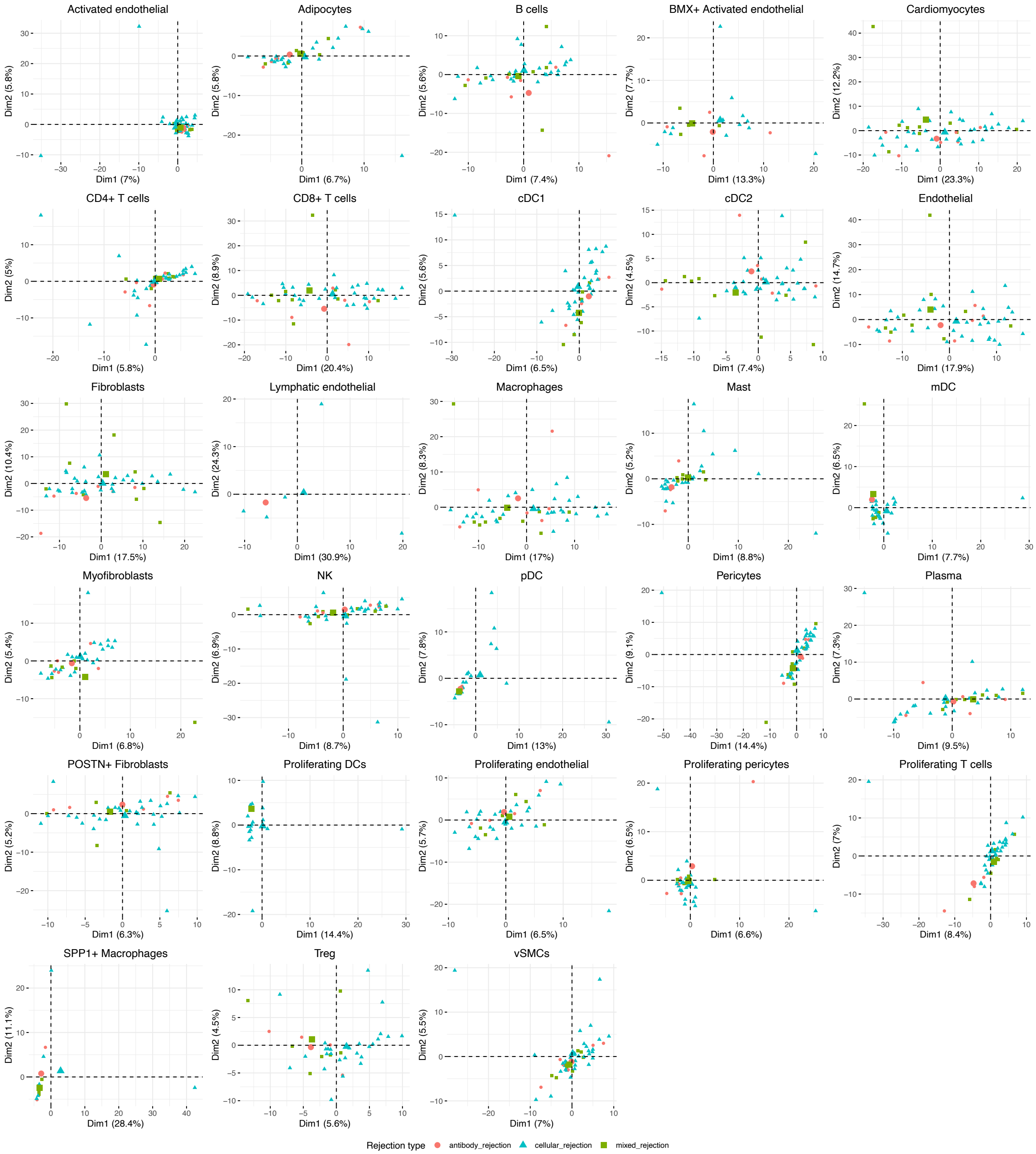

Supplementary Figure 5. Cell-specific principal components analysis (PCA) plot of gene expression patterns (x-axis: PC1; y-axis: PC2) based on pathologist-determined clinical grading.

Slide 1

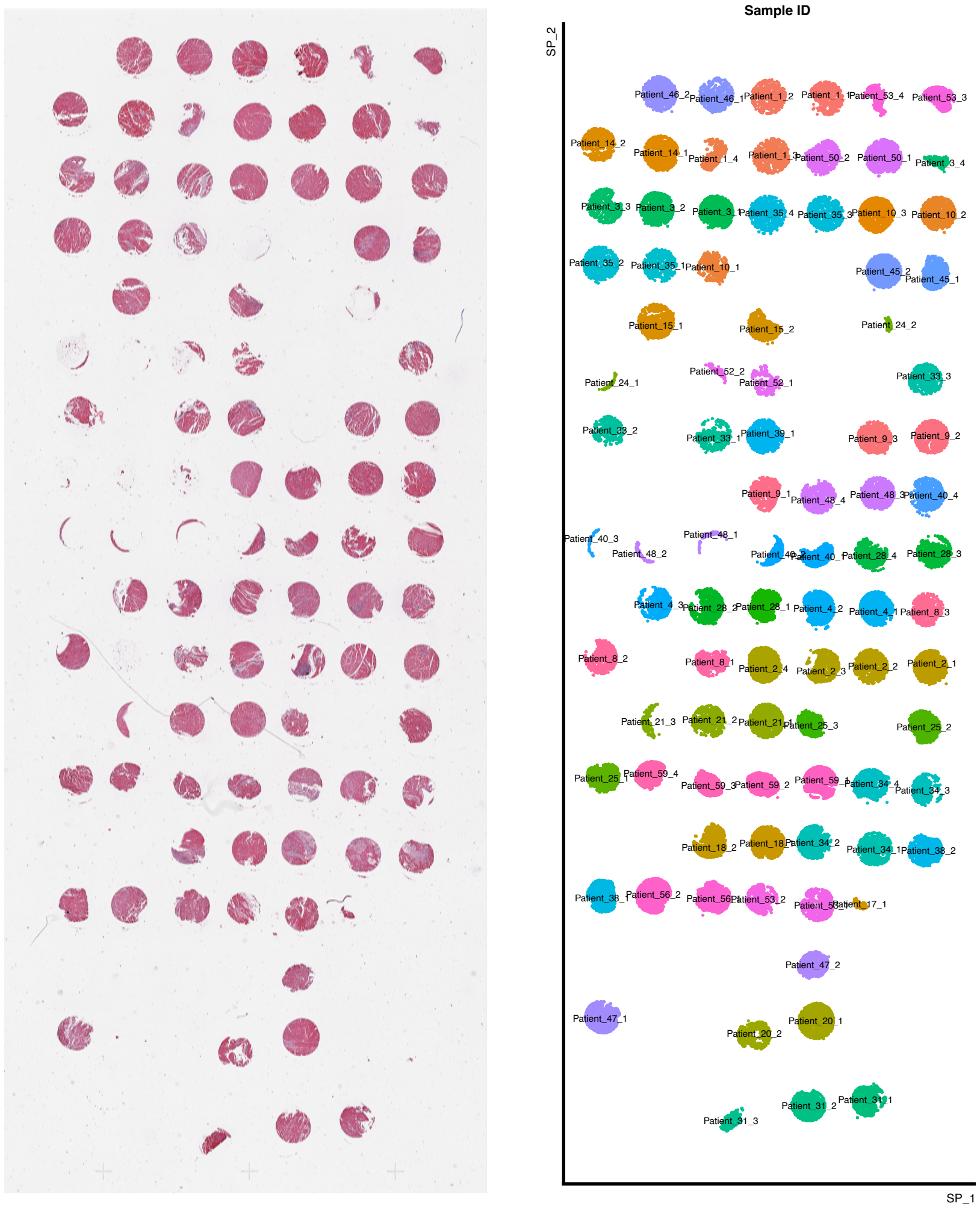

Supplementary Figure 6. H&E slide 1 image of TMAs (left) and corresponding sample ID (right).

Slide 2

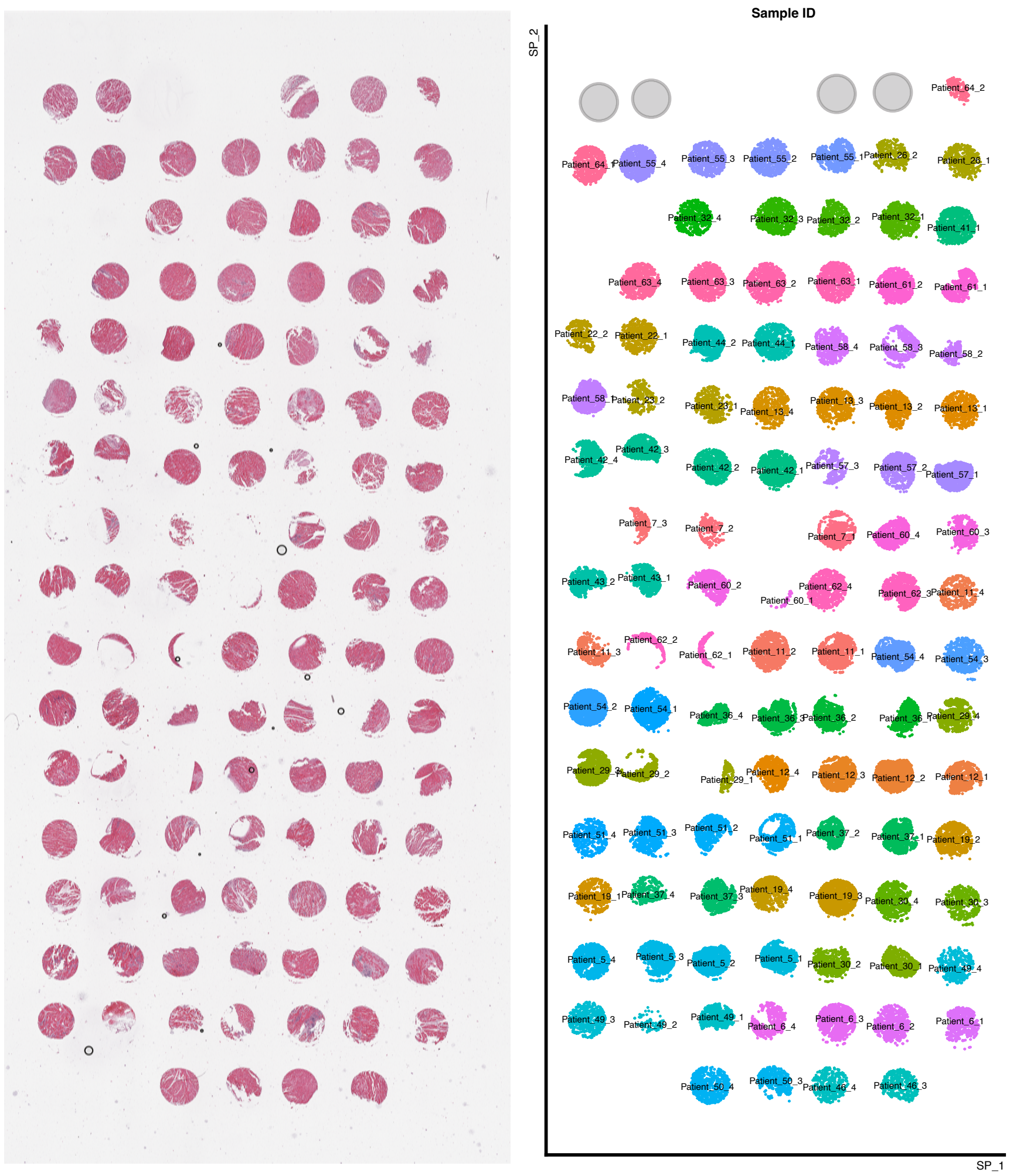

Supplementary Figure 7. H&E slide 2 image of TMAs (left) and corresponding sample ID (right).

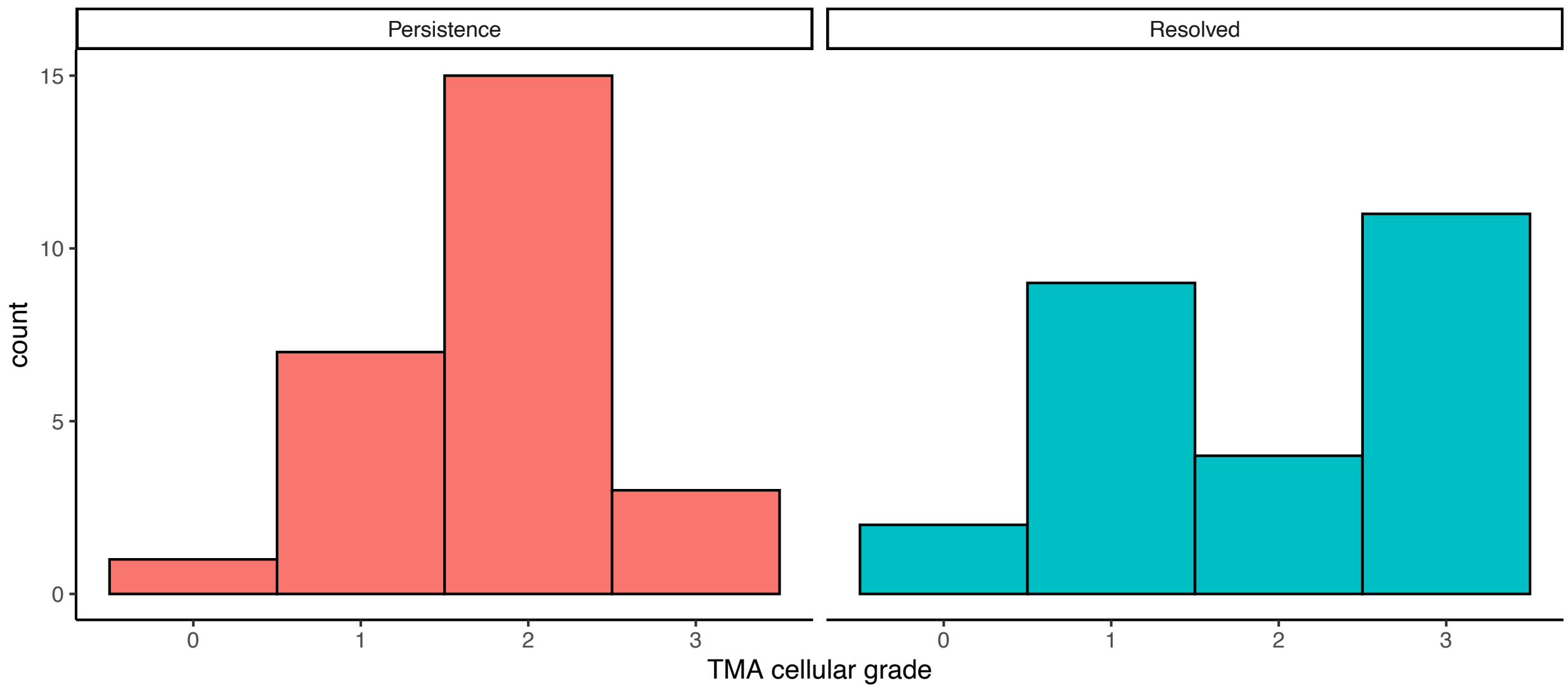

Supplementary Figure 8. Histograms of baseline TMA-level ACR grades assigned by an experienced cardiovascular transplant pathologist (R.D.H.) in those who subsequently were diagnosed with persistent (left) or resolved (right) histological ACR following anti-rejection therapies.
