# Supplementary material for: Dynamic cellular programs of human cardiac allograft rejection revealed by spatial transcriptomics
Source: Nat Cardiovasc Res. 2026 Aug 10;5(9):819–34. doi: 10.1038/s44161-026-00849-9 (PMC13561861; doi:10.1038/s44161-026-00849-9)
Supplement: Supplementary file 2 [file 44161_2026_849_MOESM2_ESM.pdf]

nature portfolio

Corresponding author(s): Nicholas Banovich and Ravi V. Shah

Last updated by author(s): Jun 19, 2026

Reporting Summary

Nature Portfolio wishes to improve the reproducibility of the work that we publish. This form provides structure for consistency and transparency in reporting. For further information on Nature Portfolio policies, see our Editorial Policies and the Editorial Policy Checklist.

Statistics

For all statistical analyses, confirm that the following items are present in the figure legend, table legend, main text, or Methods section.

n/a	Confirmed	
☐	☒	The exact sample size (*n*) for each experimental group/condition, given as a discrete number and unit of measurement
☐	☒	A statement on whether measurements were taken from distinct samples or whether the same sample was measured repeatedly
☐	☒	The statistical test(s) used AND whether they are one- or two-sided<br>*Only common tests should be described solely by name; describe more complex techniques in the Methods section.*
☐	☒	A description of all covariates tested
☐	☒	A description of any assumptions or corrections, such as tests of normality and adjustment for multiple comparisons
☐	☒	A full description of the statistical parameters including central tendency (e.g. means) or other basic estimates (e.g. regression coefficient) AND variation (e.g. standard deviation) or associated estimates of uncertainty (e.g. confidence intervals)
☐	☒	For null hypothesis testing, the test statistic (e.g. *F*, *t*, *r*) with confidence intervals, effect sizes, degrees of freedom and *P* value noted<br>*Give P values as exact values whenever suitable.*
☒	☐	For Bayesian analysis, information on the choice of priors and Markov chain Monte Carlo settings
☒	☐	For hierarchical and complex designs, identification of the appropriate level for tests and full reporting of outcomes
☒	☐	Estimates of effect sizes (e.g. Cohen's *d*, Pearson's *r*), indicating how they were calculated

Our web collection on statistics for biologists contains articles on many of the points above.

Software and code

Policy information about availability of computer code

Data collection	Xenium instrument version 2.0.1.0<br>Xenium Explorer 3.0.0
Data analysis	GitHub code link: https://github.com/Banovich-Lab/Spatial_Heart_Rejection/<br><br>ImageJ 2.14.0<br>RStudio 2023.06.0+421 "Mountain Hydrangea" Release and RStudio 2024.04.2+764 "Chocolate Cosmos" Release<br>R 4.3.0-4 and 4.4.0-3<br>Python 3.10.13<br>Seurat 5.0.1<br>Scanpy v1.8.1<br>RAPIDS v21.8.1<br>Presto 1.0.0<br>QuPath130 v0.6.0<br>Speckle 1.2.0<br>Factoextra 1.0.7.999<br>glmmTMB 1.1.7

For manuscripts utilizing custom algorithms or software that are central to the research but not yet described in published literature, software must be made available to editors and reviewers. We strongly encourage code deposition in a community repository (e.g. GitHub). See the Nature Portfolio guidelines for submitting code & software for further information.

Data

Policy information about availability of data

All manuscripts must include a data availability statement. This statement should provide the following information, where applicable:

- Accession codes, unique identifiers, or web links for publicly available datasets
- A description of any restrictions on data availability
- For clinical datasets or third party data, please ensure that the statement adheres to our policy

Raw and processed data are deposited on GEO under accession GSE290577. These include high resolution H&E and immunohistochemistry images.

Research involving human participants, their data, or biological material

Policy information about studies with human participants or human data. See also policy information about sex, gender (identity/presentation), and sexual orientation and race, ethnicity and racism.

Reporting on sex and gender	Self-reported gender was available for all 62 patients in the study. Of these patients, 21 patients were female and 41 were male.
Reporting on race, ethnicity, or other socially relevant groupings	Self-reported race/ethnicity was available for all 62 patients in the study. Of these patients, 46 were reported as white, 14 as black, 1 as white and Hispanic, and 1 as Hispanic.
Population characteristics	Of the 62 patients in this study, 42 patients were initially diagnosed with cellular rejection, 7 had antibody mediated rejection, and 13 had mix rejection. Additionally, 37 patients had persistent acute rejection after treatment and 24 had resolution after treatment. One patient did not have post-treatment data so was not determined for that patient. Ages ranged from 1-73 years.
Recruitment	*Describe how participants were recruited. Outline any potential self-selection bias or other biases that may be present and how these are likely to impact results.*
Ethics oversight	This study was approved by the Vanderbilt University Medical Center Institutional Review Board (IRB #200551)

Note that full information on the approval of the study protocol must also be provided in the manuscript.

Field-specific reporting

Please select the one below that is the best fit for your research. If you are not sure, read the appropriate sections before making your selection.

☒ Life sciences ☐ Behavioural & social sciences ☐ Ecological, evolutionary & environmental sciences

For a reference copy of the document with all sections, see nature.com/documents/nr-reporting-summary-flat.pdf

Life sciences study design

All studies must disclose on these points even when the disclosure is negative.

Sample size	The final sample size was 195 biopsies from 62 heart transplant patients (13 pediatric and 49 adults). 42 of these patients initially had cellular rejection, 7 had antibody mediated rejection, and 13 had mixed rejection. No sample size calculations were performed. We aimed to select two biopsies per-patient and per-time point (pre- and post-treatment) - one representing typical myocardium and another biopsy more representative of the condition. This dataset is currently the largest image-based spatial transcriptomic study of human heart from heart rejection patients and was sufficient to generate a large dataset of 162,638 cells.
Data exclusions	We removed cells based on standard QC filtering parameters. Cells that were too small or large, or had low number of counts were excluded from analysis.
Replication	We validated key proteins by performing immunohistochemistry (IHC) on whole-slide sections from an independent set of formalin-fixed paraffin-embedded heart biopsies: 5 non-rejection, 5 ACR (grade 2R), and 5 AMR (a mix of pAMR2 and pAMR1-i).
Randomization	*Describe how samples/organisms/participants were allocated into experimental groups. If allocation was not random, describe how covariates were controlled OR if this is not relevant to your study, explain why.*
Blinding	Investigators were not blind to the groups since acute rejection type and grade can be observed by differences in histology. Thus, blinding was not possible in this study.

Reporting for specific materials, systems and methods

We require information from authors about some types of materials, experimental systems and methods used in many studies. Here, indicate whether each material, system or method listed is relevant to your study. If you are not sure if a list item applies to your research, read the appropriate section before selecting a response.

Materials & experimental systems

n/a	Involved in the study
☒	☐ Antibodies
☒	☐ Eukaryotic cell lines
☒	☐ Palaeontology and archaeology
☒	☐ Animals and other organisms
☒	☐ Clinical data
☒	☐ Dual use research of concern
☒	☐ Plants

Methods

n/a	Involved in the study
☒	☐ ChIP-seq
☒	☐ Flow cytometry
☒	☐ MRI-based neuroimaging

Plants

Seed stocks	*Report on the source of all seed stocks or other plant material used. If applicable, state the seed stock centre and catalogue number. If plant specimens were collected from the field, describe the collection location, date and sampling procedures.*
Novel plant genotypes	*Describe the methods by which all novel plant genotypes were produced. This includes those generated by transgenic approaches, gene editing, chemical/radiation-based mutagenesis and hybridization. For transgenic lines, describe the transformation method, the number of independent lines analyzed and the generation upon which experiments were performed. For gene-edited lines, describe the editor used, the endogenous sequence targeted for editing, the targeting guide RNA sequence (if applicable) and how the editor was applied.*
Authentication	*Describe any authentication procedures for each seed stock used or novel genotype generated. Describe any experiments used to assess the effect of a mutation and, where applicable, how potential secondary effects (e.g. second site T-DNA insertions, mosiacism, off-target gene editing) were examined.*
